# Diarylthiophenes as inhibitors of the pore-forming protein perforin

**DOI:** 10.1016/j.bmcl.2015.12.003

**Published:** 2016-01-15

**Authors:** Christian K. Miller, Kristiina M. Huttunen, William A. Denny, Jagdish K. Jaiswal, Annette Ciccone, Kylie A. Browne, Joseph A. Trapani, Julie A. Spicer

**Affiliations:** a Auckland Cancer Society Research Centre, Faculty of Medical and Health Sciences, The University of Auckland, Private Bag 92019, Auckland 1142, New Zealand; b Maurice Wilkins Centre for Molecular Biodiscovery, A New Zealand Centre for Research Excellence, Auckland, New Zealand; c School of Pharmacy, Faculty of Health Sciences, University of Eastern Finland, PO Box 1627, FI-70211 Kuopio, Finland; d Cancer Immunology Program, Peter MacCallum Cancer Centre, St. Andrew’s Place, East Melbourne, Victoria 3002, Australia; e Sir Peter MacCallum Department of Oncology, The University of Melbourne, Parkville, Victoria 3052, Australia

**Keywords:** HTS, high-throughput screen, SAR, structure–activity relationships, PRF, perforin, CTL, cytotoxic T lymphocytes, NK, natural killer cells, PAINS, pan-assay interference compounds, AgNO_3_, silver(I) nitrate, KF, potassium fluoride, PFP, pentafluorophenyl, DCC, *N*,*N*′-dicyclohexylcarbodiimide, HOBt, hydroxybenzotriazole, EDCI, 1-ethyl-3-(3-dimethylaminopropyl)carbodiimide, DMF, dimethylformamide, KOAc, potassium acetate, DMSO, dimethyl sulfoxide, THF, tetrahydrofuran, Perforin, Perforin inhibitor, Diarylthiophene, Bioisostere, Immunosuppressant

## Abstract

Evolution from a furan-containing high-throughput screen (HTS) hit (**1**) resulted in isobenzofuran-1(3*H*)-one (**2**) as a potent inhibitor of the function of both isolated perforin protein and perforin delivered in situ by intact KHYG-1 NK cells. In the current study, structure–activity relationship (SAR) development towards a novel series of diarylthiophene analogues has continued through the use of substituted-benzene and -pyridyl moieties as bioisosteres for 2-thioxoimidazolidin-4-one (**A**) on a thiophene (**B**) -isobenzofuranone (**C**) scaffold. The resulting compounds were tested for their ability to inhibit perforin lytic activity in vitro. Carboxamide (**23**) shows a 4-fold increase over (**2**) in lytic activity against isolated perforin and provides good rationale for continued development within this class.

## Graphical abstract

**Figure:**
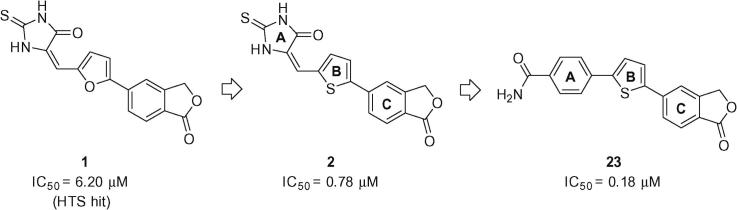

Perforin (PRF) is a calcium-dependent pore-forming glycoprotein found within granules of cytotoxic T lymphocytes (CTL) and natural killer (NK) cells.^1^, ^2^ These cytotoxic effector cells play an important role in immune-surveillance and homeostasis via a granule exocytosis pathway responsible for the elimination of neoplastic/virus-infected cells and intracellular pathogens.^3^ Numerous autoimmune diseases (e.g., insulin-dependent diabetes) and therapy-induced conditions (e.g., allograft transplantation rejection, graft-vs-host disease) adopt a synonymous biochemical pathway.^4^, ^5^ An integral part of this biological cascade is perforin oligomerisation in which monomeric perforin binds calcium upon entry into the immune synaptic cleft and forms highly ordered oligomers with 19–24 adjacent perforin monomers. These in turn form cylindrical-type pores which subsequently penetrate target cell membranes, allowing rapid delivery of cytotoxic granzymes into the cytosolic compartment, a process which culminates in apoptosis of the target cell. Highly specific inhibitors of perforin function are thus of interest as selective immunosuppressive drugs.

Isobenzofuran-1(3*H*)-one-based inhibitor **1** (Fig. 1) was a hit selected from a high-throughput screen of approximately 100,000 compounds sourced from commercial ‘lead discovery’ libraries.^6^ Subsequent SAR development led to the discovery of potent inhibitor **2** in which an ∼8-fold improvement in inhibition of isolated perforin protein function was achieved through replacement of a central furan ring with a 2,5-thiophene moiety.^7^ In more recently published work^8^ this template was elaborated further through variation of the isobenzofuranone C-subunit where an extensive SAR programme was conducted and the mechanism of perforin inhibition investigated using real-time microscopy.^8^, ^9^ The resulting isoindolinone-containing inhibitors were shown to have improved lytic activity against perforin delivered in situ by intact KHYG-1 NK cells and furthermore, were employed as probes to elucidate the role of perforin in the granule exocytosis pathway. This work resulted in the conclusion that this class of compounds target perforin after its release into the immune synapse.

While substantial advances have been made in the development and understanding of the thioxoimidazolidinone-based series, more detailed in vitro and in vivo assessment^7^, ^10^ revealed several properties requiring further optimisation. Although well-tolerated in healthy mice, even those compounds deemed suitable candidates for further in vivo studies showed some toxicity toward whole NK cells.^8^ It is therefore likely that toxicity would also be observed in the immunocompromised mice required for an efficacy study. Also desirable would be the identification of new compounds with significantly improved potency to deliver a lower efficacious dose and better control of potential dose-related toxicity. Finally, while the thioxoimidazolidinone series passed various pan-assay interference compound (PAINS) filters,^11^ they still contain a potentially reactive Michael acceptor and exist as an inseparable (and interconverting) mixture of *E*- and *Z*-isomers.

To address these aforementioned issues the need for a 2-thioxoimidazolidin-4-one bioisosteric replacement was apparent and herein we report on the latest developments towards an exciting new class of nontoxic inhibitors of perforin function that show improved inhibition of the function of both isolated perforin protein and perforin delivered in situ by intact KHYG-1 NK cells.

*Synthesis*: Target compounds **6**–**23** (Scheme 1) were obtained in a linear four-step sequence starting with the conversion of commercially available 2-methyl-4-bromobenzoic acid to 5-bromoisobenzofuran-1(3*H*)-one **3**.^12^ A sequential Finkelstein halogen exchange reaction^13^ gave 5-iodoisobenzofuran-1(3*H*)-one **4** in 87% yield. This can also be prepared in high yield through diazotization of the corresponding 5-aminoisobenzofuran-1(3*H*)-one using a literature procedure.^14^ Lactone derivative **4** was then utilised in a palladium-catalysed arylation reaction with 2-bromothiophene in the presence of a silver(I) nitrate/potassium fluoride (AgNO_3_/KF) activator system^15^ to yield key intermediate bromide **5**. Subsequent Suzuki couplings with a variety of commercially available phenyl-boronates/boronic acids in the presence of catalytic PdCl_2_(dppf), afforded the respective diarylthiophene targets **6**–**23** in generally good yields. Isoindolinone-based versions of **23** (**23a** and **23b**) were also prepared in exactly the same manner, using the isoindolin-1-one and 2-methylisoindolin-1-one-based analogues^16^ of key bromide **5** (**5a** and **5b**; Scheme 1).

Analogous methodology was adopted to generate secondary and tertiary carboxamide targets **29** and **30**, by first reacting iodide **24** with 2-bromothiophene in the presence of AgNO_3_/KF (Scheme 2). The afforded bromide **25** was then converted to carboxamide intermediates **26** and **27**^17^ through activation of the carboxylic acid to a pentafluorophenyl (PFP) ester followed by subsequent addition of the corresponding amine (methylamine or dimethylamine) in pyridine for 2–3 h at room temperature. Key boronate **28** was prepared in multi-gram quantities from bromide **3**^18^ (Scheme 1) and subsequently used in a Suzuki coupling with intermediates **26** and **27** to furnish carboxamide targets **29** (75%) and **30** (57%), respectively.

Compounds **31** and **32**, where the carboxamide side-chain was elaborated further by incorporating a series of basic side-chains, were accessed by applying the same chemistry as summarised in Scheme 1. A final step Suzuki coupling of key intermediate bromide **5** with the corresponding phenyl boronate (as according to the compounds of Table 1) gave **31** and **32** in good to modest yields, respectively, (61% and 42%).

With the chemistry now established for the mono-substituted A-ring targets shown in Scheme 1, Scheme 2, a series of di-substituted targets were investigated. Phenyl iodides **33**–**35** were prepared via esterification from their native carboxylic acids in the presence of EtOH and catalytic H_2_SO_4_, using conventional methodology (Scheme 3). The sequential step was carried out as for carboxylic acid analogue **25** (refer to Scheme 2) affording ethyl esters **36**–**38**, these in turn were then coupled with key boronate ester **28** via a Suzuki reaction to give diarylthiophene esters **39**–**41** in good to excellent yields. Hydrolysis of the resulting esters with 2 M NaOH in MeOH under reflux conditions, gave bis-carboxylic acid intermediates **42**–**44**, respectively. Subsequent dehydration to isobenzofuran-1(3*H*)-one precursors **45**–**47** was achieved quantitatively using a mixture of TFA/CH_2_Cl_2_ (1:1) at room temperature for approximately 3–4 h. Target compounds **48**–**57** were then synthesised utilising a variety of amide-coupling strategies (Scheme 3). Carboxamide **48** was prepared successfully in 57% yield going via the analogous PFP-active ester intermediate as employed for carboxamides **26** and **27** (refer to Scheme 2). Compounds **49**–**54**, which contain various extended neutral and basic side-chains appended to the 4-carboxamide moiety, were accessed through treatment of the corresponding acid precursor with DCC and catalytic HOBt at elevated temperatures. Target compounds **55** (48%), **56** (80%), and **57** (38%), all of which contain a 3-substituted-phenyl primary amine, were accessed through an EDCI/HOBt-mediated amide-coupling in the presence of anhydrous DMF at 0 °C to room temperature over 1–2 h.

Compounds **58**–**63** (Scheme 1) encompass a variety of A-ring moieties (such as an alcohol, a methanethiol and various sulfonamides). These were all accessed through a key Suzuki coupling step from key intermediate **5** with the corresponding substituted boronate, as according to the compounds of Table 1. Isobenzofuran-1(3*H*)-one dimer (**71**) was prepared in the same manner.

Bromide **64** was commercially available and was converted to iodide **65** via a copper-mediated halogen exchange reaction as adapted from literature procedure^13^ (Scheme 4). Boronate **66** was synthesised from corresponding bromide intermediate **5** in the presence of bis(pinacolato)diboron and KOAc and subsequently coupled with iodide **65** utilising Suzuki methodology to yield pyridyl carboxamide **67** in low yield.

Finally, the preparation of 4-pyridyl-containing target **70** began with the Suzuki-coupling of 4-bromopyridine **68** with thiophene-2-boronic acid, as described by Effenberger et al.,^19^ to furnish 4-(2-thienyl)pyridine **69** in high yield. Isobenzofuran-1(3*H*)-one **4** was then installed through treatment with AgNO_3_ and KF in the presence of a palladium-complex, as previously discussed, to afford 4-pyridyl derivative **70** in 27% yield (Scheme 1).

*Structure–activity relationships*: Based on our previous studies described above, we hypothesised that selected substitutions on a benzene or pyridyl ring could potentially occupy the same space as the thioxoimidazolidinone pharmacophore, thereby mimicking the network of hydrogen bond donors/acceptors required for activity.^8^ This approach is illustrated in Figure 2 and highlights key objectives which include removal of the Michael acceptor and elimination of isomeric mixtures.

For the current study we elected to retain the isobenzofuranone C-subunit of **2** as this class is generally more potent than the corresponding isoindolinones^8^ which were originally introduced on the assumption they would be less susceptible to hydrolysis. However subsequent stability studies showed no clear advantage with both the isoindolinones and isobenzofuranones showing variable half-lives in vitro (microsome stability) and in vivo.^11^ Parent compound (**6**) was employed as a start point for the replacement of the 2-thioxoimidazolidin-4-one A-subunit, but due to its lack of potency (Jurkat IC_50_ >20 μM; Table 1) a series of simple substituted phenyl based derivatives were designed and investigated.

Compounds **7**–**15** (Table 1) follow an independent 2, 3, 4 substitution pattern on a benzene A-ring with derivative **15** (4-OMe) demonstrating activity against isolated perforin protein in our Jurkat assay (IC_50_ = 9.36 μM). The remaining methoxy isomers (**13**, **14**) showed no detectable inhibition, suggesting that substitution *para* to the connecting thiophene B-subunit is essential for activity. Prompted by this encouraging result and with derivative **15** providing an H-bond acceptor moiety, we looked to introduce an NH_2_, OH and CN group at positions 3 and 4 accordingly. Isomers **16** (3-NH_2_) and **17** (4-NH_2_) both have comparable activity (IC_50_s = 13.97 and 11.82 μM, respectively), showing that substitution at the 3-position is tolerated in this particular case. This is confirmed further by 3-hydroxyl-containing compound **18** in which a 2-fold increase in potency is achieved (IC_50_ = 5.89 μM). Looking at 3-CN derivative **20** in comparison, activity is lost altogether suggesting that an H-bond donor may be required in this position for activity. In contrast, 4-OH containing compound **19** loses all activity whilst 4-CN containing compound **21** (IC_50_ = 6.87 μM) retains potency similar to that of **18**. This reinforces the argument in that an H-bond acceptor at position 4 is more favourable and perhaps necessary for activity to exist in this series (**15** and **21** vs **19**).

Compounds **22** and **23** were designed with a carboxamide moiety installed at positions 3 and 4, respectively. Substitution at position 3 was well tolerated giving rise to an IC_50_ = 2.97 μM—2-fold greater than our previously most active compound **18**. However when the primary amide is moved to position 4, a dramatic increase in the ability to inhibit perforin lytic activity is seen. Benzene-4-carboxamide **23** has a potency approximately 4-fold greater than lead thioxoimidazolidinone **2** (Figure 1, Figure 2) against isolated perforin (0.18 μM vs 0.78 μM, respectively). Furthermore, the corresponding pyridine-4-carboxamide **67** is also one of our most potent compounds (IC_50_ = 0.92 μM). Although at the outset a decision was made to retain the more potent isobenzofuranone C-subunit, for completeness the analogous isoindolin-1-one (**23a**) and 2-methylisoindolin-1-one (**23b**) derivatives were also prepared. As expected this modification resulted in a loss of activity. Conversion of the primary amide of **23** to a secondary derivative (**29**) results in a loss of efficacy, while the presence of a tertiary amide (**30**, **31**) abolishes activity completely. Ester **39** showed limited activity (10.97 μM), while compounds **40**, **46**, **48** and **55** were designed in an effort to combine the outstanding potency of **23** with the preferred 3-OH of **18** or 3-NH_2_ of **16**, thereby introducing an H-bond donor at the 3-position and adding an ionisable centre to assist solubility. While this approach did generate potent (and slightly more soluble—see Supplementary data) compounds, none were an improvement on **23**. In an extension of this strategy, solubilising sidechains were introduced in examples **32**, **48**–**54** and **56**, **57**. Disappointingly, compounds with strongly or weakly basic sidechains (**32**, **49**, **50**, **56**, **57**) displayed poor activity or were inactive, while neutral compounds **51** and **53** showed only moderate activity (IC_50_s 3.31 and 2.65 μM), respectively.

In order to further increase diversity and enrich the SAR of the diarylthiophene series, a range of commercially available boronates were deployed in Suzuki reactions with key intermediate **5** (Scheme 1), generating analogues **58**–**63**. Results were mixed, with the methyl alcohol **59** and methyl sulfonamide **63** (IC_50_s 0.92 and 1.09 μM, respectively) the best of this set. Finally, given similarities in SAR between the A- and C-subunits^7^ the symmetrical analogue **71** was synthesised but proved only moderately potent as well as extremely insoluble.

*Biological activity and stability*: The five most potent inhibitors of isolated recombinant perforin (**23**, **48**, **59**, **63**, and **67** from Table 1) were then subjected to more advanced assessment. Preliminary stability studies were carried out by incubation in human or mouse plasma at 37 °C with the percentage parent remaining measured at 24 h (Table 2). All five inhibitors were significantly more stable in mouse plasma compared to human. This result is not unexpected, as the anticancer agent camptothecin which also contains a lactone moiety has been shown to co-exist in both the closed and ring-opened form and that this equilibrium is distinctly different for human and mouse plasma. In mouse plasma the ratio of open to closed is 50:50%, while in human plasma this shifts to 90:10 due to the strong affinity of human serum albumin for the ring-opened form.^20^ It is likely that the same phenomenon is operating in the present case and while not necessarily an issue for mouse studies, will need to be addressed in future work.

The compounds were then evaluated for their ability to block the effect of perforin delivered by whole KHYG-1 NK cells (Table 2). Employing whole NK cells is a more rigorous model of conditions in vivo than the use of isolated recombinant protein since effector cell identification of the labelled target cell and formation of an immune synapse are required for perforin release. Any putative inhibitor must also possess the ability to access perforin released into the synaptic cleft. After co-incubation of inhibitor with KHYG-1 NK cells in medium for 30 min at room temperature, ^51^Cr-labelled K562 leukaemia target cells were added and cell lysis evaluated after 4 h incubation at 37 °C by measuring ^51^Cr release. The viability of the NK cells in the presence of inhibitor was also assessed 24 h. later to confirm that any inhibitory activity exhibited by the compounds was due to blocking the action of perforin and not nonspecific killing of the effector cells. Viable and dead cells were counted and percent viability calculated based on total cell count (for further details see Supplementary data). Of the five compounds selected for further testing, three showed poor or no activity; the 3-OH, 4-CONH_2_ compound **48**, 4-methyl alcohol **59** and methanesulfonamide **63**. Gratifyingly, the most potent compound against isolated perforin (**23**) showed comparable activity (60% inhibition of lytic activity at 20 μM concentration) to many of the active compounds contained in our previous report on the thioxoimidazolidinone-based series.^8^ In addition, pyridinecarboxamide **67** was essentially equivalent to our previously most potent in vivo candidates (80% inhibition of lysis). Most importantly, this activity was observed in the absence of toxicity toward the effector cells (90% and 82% viability, respectively). For the purposes of this work, compounds are classified as toxic if NK cell viability falls below 70%. This is due to the importance of CTLs and NK cells in the overall immunological response which means that it is essential that any pharmacological intervention allows rapid recovery of these cytotoxic effector cells for normal immunological function.

In summary a new series of benzene- and pyridine-carboxamides have been designed as isosteric replacements for our previous thioxoimidazolidinone-based series. Several of these compounds showed improved inhibition of perforin-mediated lysis, with benzenecarboxamide **23** demonstrating a 4-fold increase (0.18 μM) over the corresponding thioxoimidazolidinone inhibitor **2** (0.78 μM). Compound **23** and its pyridine analogue **67** also showed excellent activity against the lytic action of whole human NK cells, at a level comparable to our previously identified in vivo candidates.^8^ Use of an isostere in place of the thioxoimidazolidinone A-subunit has also enabled removal of a potentially reactive Michael acceptor moiety and elimination of *E*- and *Z*-isomeric mixtures. Finally, these compounds do not appear to possess the marginal toxicity associated with many of the thioxoimidazolidinones.

We can conclude that we have identified viable replacements for the thioxoimidazolidinone subunit while improving potency. We have also overcome several key concerns in relation to this historic series and now seek to expand on these findings to identify a suitable in vivo candidate. The mechanism of action of these inhibitors is still under investigation, however given our previous conclusion that this class of compounds target perforin after its release into the immune synapse it is most likely that inhibition occurs by either (1) blocking the monomers from association with the target cell membrane, (2) counteracting the oligomerisation process that forms the pre-pore, or (3) by blocking of the conformational changes required for pore formation. This work is currently ongoing and further developments will be reported in the future.

## Figures and Tables

**Figure 1:**
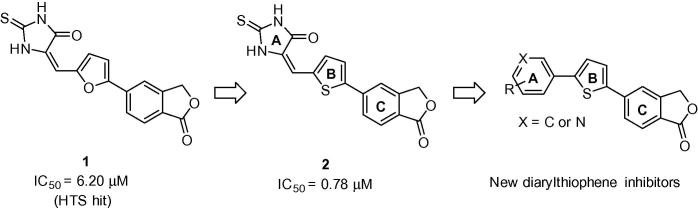
The development of new diarylthiophene inhibitors of perforin.

**Figure 2:**
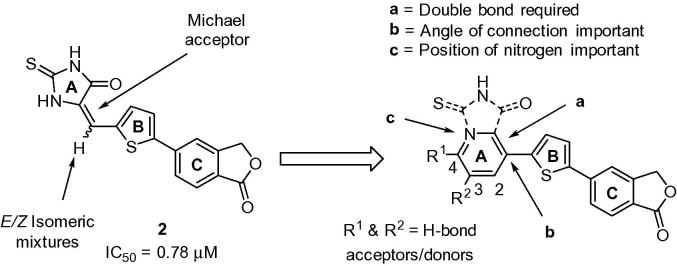
Rationale for the development of new diarylthiophene inhibitors.

**Scheme 1:**
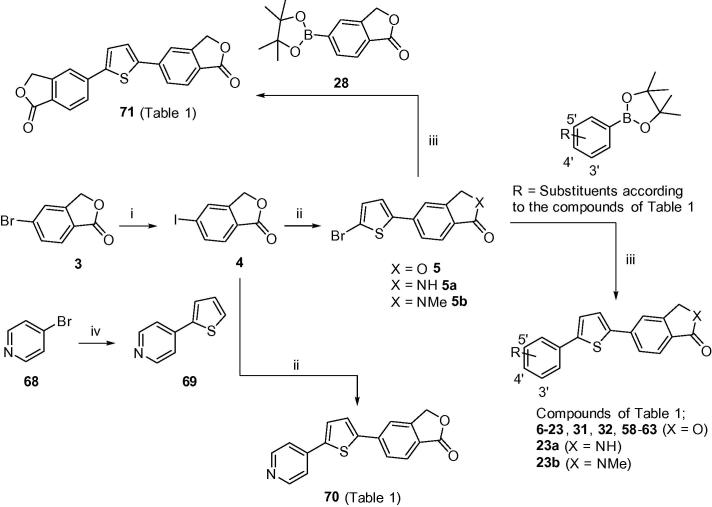
Reagents and conditions: (i) sealed tube, 1,4-dioxane, CuI (5.0 mol %), NaI, racemic *trans*-*N*,*N*′-dimethyl-1,2-cyclohexanediamine (10 mol %), 110 °C, N_2_, 18 h; (ii) 2-bromothiophene or **69**, DMSO, KF, PdCl_2_(PPh_3_)_2_, AgNO_3_, 100 °C, N_2_, 2 h; (iii) toluene/EtOH, 2 M Na_2_CO_3_, PdCl_2_(dppf)·CH_2_Cl_2_, 85–90 °C, N_2_, 2–6 h; (iv) thiophene-2-boronic acid, toluene/EtOH, 2 M Na_2_CO_3_, PdCl_2_(dppf)·CH_2_Cl_2_, 85 °C, N_2_, 2 h.

**Scheme 2:**
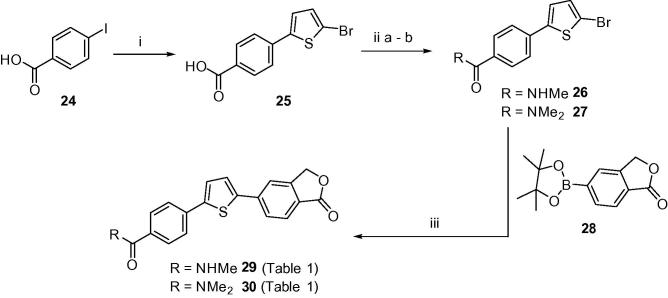
Reagents and conditions: (i) 2-bromothiophene, DMSO, KF, PdCl_2_(PPh_3_)_2_, AgNO_3_, 100 °C, N_2_, 2 h; (ii) (a) THF, pyridine, pentafluorophenyltrifluoroacetate, rt, 2 h; (b) THF, methylamine (40% soln.) or dimethylamine, rt, 2–3 h; (iii) toluene/EtOH, 2 M Na_2_CO_3_, PdCl_2_(dppf)·CH_2_Cl_2_, 85–90 °C, N_2_, 2–6 h.

**Scheme 3:**
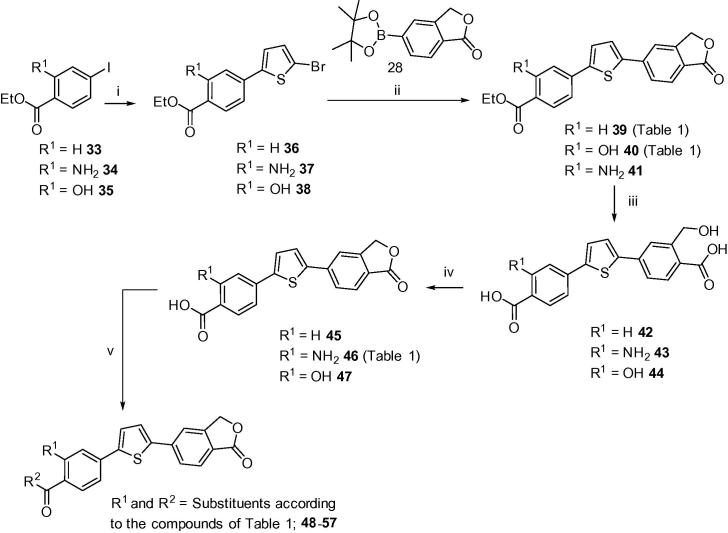
Reagents and conditions: (i) 2-bromothiophene, DMSO, KF, PdCl_2_(PPh_3_)_2_, AgNO_3_, 100 °C, N_2_, 2–4 h; (ii) toluene/EtOH, 2 M Na_2_CO_3_, PdCl_2_(dppf)·CH_2_Cl_2_, 85–90 °C, N_2_, 1–6 h; (iii) MeOH, 2 M NaOH, 100 °C, 2 h, HCl-workup; (iv) TFA–DCM (1:1), rt, 3–4 h; (v) THF, pyridine, pentafluorophenyltrifluoroacetate, rt, 2 h, R^2^-NH_2_, rt, 2 h, **48**; or pyridine, DCC, HOBt, 75 °C, 5–6 h, **49–54**; or anhydrous-DMF, EDCI·HCl, HOBt, rt, 2 h, R^2^-NH_2_, 0 °C → rt, 1–2 h, **55**–**57**.

**Scheme 4:**
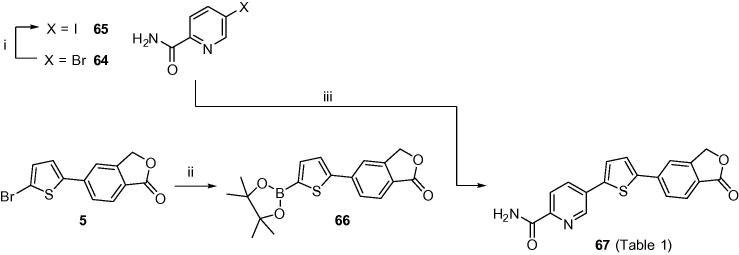
Reagents and conditions: (i) sealed tube, 1,4-dioxane, CuI (5.0 mol %), NaI, racemic *trans*-*N*,*N*′-dimethyl-1,2-cyclohexanediamine (10 mol %), 110 °C, N_2_, 24 h; (ii) DMSO, bis(pinacolato)diboron, KOAc, PdCl_2_(dppf)·CH_2_Cl_2_, 90 °C, N_2_, 4 h; (iii) toluene/EtOH, 2 M Na_2_CO_3_, PdCl_2_(dppf)·CH_2_Cl_2_, 85–90 °C, N_2_, 1.5 h.

**Table 1:** Inhibitory activities of isobenzofuran-1(3*H*)-ones with various A-subunits

**Figure:**
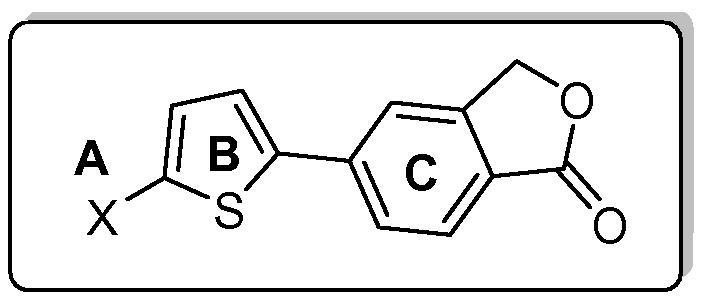

Number	Scheme	X^a^	Inhibition of Jurkat cell lysis IC_50_^b^ (μM)
**6**	1	Ph	>20
**7**	1	2-Me Ph	>20
**8**	1	3-Me Ph	>20
**9**	1	4-Me Ph	>20
**10**	1	2-Cl Ph	>20
**11**	1	3-Cl Ph	>20
**12**	1	4-Cl Ph	>20
**13**	1	2-OMe Ph	>20
**14**	1	3-OMe Ph	>20
**15**	1	4-OMe Ph	9.36
**16**	1	3-NH_2_ Ph	13.97
**17**	1	4-NH_2_ Ph	11.82
**18**	1	3-OH Ph	5.89
**19**	1	4-OH Ph	>20
**20**	1	3-CN Ph	20
**21**	1	4-CN Ph	6.87
**22**	1	3-CONH_2_ Ph	2.97
**23**	1	4-CONH_2_ Ph	0.18
**23a**^c^	1	4-CONH_2_ Ph	6.04
**23b**^d^	1	4-CONH_2_ Ph	>20
**29**	2	4-CONHMe Ph	10.7
**30**	2	4-CONMe_2_ Ph	>20
**31**^e^	1		>20
**32**	1	4-CONH(CH_2_)_3_NMe_2_ Ph	>20
**39**	3	4-COOEt Ph	10.97
**40**	3	3-OH, 4-COOEt Ph	>20
**46**	3	3-NH_2_, 4-COOH Ph	5.84
**48**	3	3-OH, 4-CONH_2_ Ph	0.67
**49**^e^	3		12.75
**50**^e^	3	3-OH, 4-CONH(CH_2_)_2_NMe_2_ Ph	>20
**51**	3	3-OH, 4-CONHCH_2_CH_2_OH Ph	3.31
**52**	3	3-OH, 4-CONHCH_2_CH(OH)CH_3_ Ph	>20
**53**	3	3-OH, 4-CONHCH_2_CONH_2_ Ph	2.65
**54**	3	3-OH, 4-CONHCH_2_CH_2_CONH_2_ Ph	7.12
**55**	3	3-NH_2_, 4-CONH_2_ Ph	1.20
**56**^e^	3		14.71
**57**^e^	3	3-NH_2_, 4-CONH(CH_2_)_2_NMe_2_ Ph	>20
**58**	1	4-SMe Ph	>20
**59**	1	4-CH_2_OH Ph	0.90
**60**	1	4-SO_2_Me Ph	>20
**61**	1	3-SO_2_NH_2_ Ph	4.85
**62**	1	4-SO_2_NH*tert-*Bu Ph	6.09
**63**	1	4-NHSO_2_Me Ph	1.09
**67**	4	4-CONH_2_, 3-pyridyl	0.92
**70**	1	4-Pyridyl	4.53
**71**	1	5-Isobenzofuran-1(3*H*)-one	6.24

a Structure of the A-subunit.

b Testing was carried out over a range of doses, with the IC_50_ being equal to the concentration at which 50% inhibition of the lysis of Jurkat cells by perforin was observed, as measured by ^51^Cr release. Values are the average of at least two independent IC_50_ determinations.

c Isobenzofuran-1(3*H*)-one C-subunit replaced with an isoindolin-1-one.

d Isobenzofuran-1(3*H*)-one C-subunit replaced with an 2-methylisoindolin-1-one.

e Detailed structures for compounds, **31**, **49**, **50**, **56** and **57** can be found in the Supplementary information (Experimental Section).

**Table 2:** Capacity of selected compounds to inhibit perforin delivered by KHYG-1 NK cells

Number	Jurkat IC_50_^a^ (μM)	KHYG-1 inhibition^b^ (% at 20 μM)	KHYG-1 viability^c^ (%)	Plasma stability^d^ (% at 24 h)	
Mouse	Human
**23**	0.18	63	90	75	46
**48**	0.67	20	92	75	49
**59**	0.90	13	79	87	73
**63**	1.09	No activity	—	80	70
**67**	0.92	80	82	66	39

a Data given for five most potent compounds as determined by the Jurkat assay.

b Inhibition by compound (20 μM) of the perforin-induced lysis of K562 target cells when co-incubated with KHYG-1 human NK cells (see Supplementary data). Percent inhibition calculated compared to untreated control.

c Viability of KHYG-1 NK cells after 24 h by Trypan blue exclusion assay (see Supplementary data).

d Data given as % parent measured at 24 h in mouse and human plasma (see Supplementary data for further details).

## Supplementary data

Supplementary data Experimental procedures.
