# Supplementary material for: Diarylthiophenes as inhibitors of the pore-forming protein perforin
Source: Bioorg Med Chem Lett. 2016 Jan 15;26(2):355–60. doi: 10.1016/j.bmcl.2015.12.003 (PMC4706532; doi:10.1016/j.bmcl.2015.12.003)
Supplement: Supplementary data — Experimental procedures. [file mmc1.docx]

**SUPPLEMENTRY MATERIAL**

**Diarylthiophenes as inhibitors of the pore-forming protein perforin**

Christian K. Miller, Kristiina M. Huttunen, William A. Denny, Jagdish K. Jaiswal, Annette Ciccone, Kylie A. Browne, Joseph A. Trapani, Julie A. Spicer^*^

*Corresponding author:* ^*^Tel.: +64 9 3737599; Fax: +64 9 3737502

E-mail: j.spicer@auckland.ac.nz

**CONTENTS**:

1. Experimental Page 2

1.1. Chemistry Page 2

1.1.1. Table 1: Elemental analysis results for target compounds Page 15

1.1.2. Table 2: Elemental analyses for selected intermediates Page 16

1.1.3. Table 3: HRMS and HPLC results for target compounds Page 16

1.1.4. Table 4: Solubility data for selected compounds Page 16

1.2. Biology Page 16

1.2.1 Inhibition of perforin-mediated lysis of sheep red blood cells Page 16

1.2.2 Inhibition of perforin-mediated lysis of Jurkat cells Page 17

1.2.3 KHYG-1 Cytotoxicity Assay Page 17

1.2.4 Toxicity to KHYG-1 NK Cells Page 17

2. Plasma stability studies Page 18

3. References Page 18

**1. Experimental**

**1.1. Chemistry**

Analyses were performed by the Microchemical Laboratory, University of Otago, Dunedin, NZ. Melting points were determined using an Electrothermal Model 9200 and are as read. NMR spectra were measured on a Bruker Advance 400 MHz spectrometer and referenced to Me_4_Si. Mass spectra were recorded either on a Varian VG 7070 spectrometer at nominal 5000 resolution or a Finnigan MAT 900Q spectrometer. All final compound purities were determined to be >95% by HPLC on an Alltech Alltima C18 column (3.2 x 150 mm, 5 μm) eluting with 5–80% MeCN/45 mM NH_4_HCO_3_.

**General Procedure A: 5-(5-Bromothiophen-2-yl)isobenzofuran-1(3*H*)-one (5).** Compound **4**^14^ and 2-bromothiophene were reacted using a procedure adapted from a literature reference.^15^ The iodide (500 mg, 1.92 mmol), PdCl_2_(PPh_3_)_2_ (67 mg, 0.10 mmol) and KF (224 mg, 3.85 mmol) were weighed into a flask and dissolved in DMSO (6 mL). The mixture was placed under an atmosphere of N_2_, 2-bromothiophene (376 mg, 2.31 mmol) and AgNO_3_ (163 mg, 0.96 mmol) added, then the resulting suspension stirred for 0.5 h at 100 ^o^C. Further portions of AgNO_3_ (3x163 mg) were added at 0.5 h. intervals, to give a total reaction time of 2 h. Upon cooling, the mixture was filtered through a plug of celite which was washed well with CHCl_3_. The resulting CHCl_3_ (ca 200 mL) solution was washed with water (3x100 mL), dried (Na_2_SO_4_) and the solvent removed under reduced pressure to give a crude solid which was purified by flash column chromatography on silica gel (20% EtOAc/hexanes as eluant). The title compound **5** was isolated as a crystalline yellow solid (478 mg, 84%), m.p. (EtOAc/hexanes) 181-183 ^o^C. ^1^H NMR [(CD_3_)_2_SO] δ 7.91 (s, 1 H), 7.87 (dd, *J* = 0.6, 8.0 Hz, 1 H), 7.84 (dd, *J* = 1.4, 8.0 Hz, 1 H), 7.59 (d, *J* = 3.9 Hz, 1 H), 7.35 (d, *J* = 3.9 Hz, 1 H), 5.44 (s, 2 H). LRMS (APCI^+^) calcd for C_12_H_8_BrO_2_S 295 (MH^+^), found 295. Anal. (C_12_H_7_BrO_2_S.0.1EtOAc) C, H.

**General Procedure B: Ethyl 4-(5-(1-oxo-1,3-dihydroisobenzofuran-5-yl)thiophen-2-yl)benzoate (39).** Compound **36** (230 mg, 0.74 mmol; prepared according to a literature procedure^15^) was dissolved in toluene (6.0 mL), to which was added a suspension of **28** (192 mg, 0.74 mmol) in EtOH (3.0 mL). A solution of Na_2_CO_3_ (2 M, 1.5 mL) and Pd(dppf)Cl_2_ (30 mg, 0.04 mmol) were added and the entire mixture heated at reflux under N_2_ for 1 h. Upon cooling, the mixture was diluted with water and extracted with CH_2_Cl_2_ (10x50 mL). The combined organic fractions were dried (Na_2_SO_4_), filtered, and the solvent removed under reduced pressure to give a crude solid which was purified by flash column chromatography on silica gel (CH_2_Cl_2_ as eluant). The title compound **39** was isolated as a pale yellow solid (55%), mp (Et_2_O) 199-201 ^o^C. ^1^H NMR [(CD_3_)_2_SO] δ 8.02 (m, 3 H), 7.94 (d, *J* = 8.1 Hz, 1 H), 7.88 (m, 3 H), 7.82 (d, *J* = 3.9 Hz, 1 H), 7.79 (d, *J* = 3.91 Hz, 1 H), 5.45 (s, 2 H), 4.34 (q, *J* = 7.1 Hz, 2 H), 1.34 (t, *J* = 7.1 Hz, 3 H). LRMS (APCI^+^) calcd for C_21_H_17_O_4_S 365 (MH^+^), found 365. Anal. (C_21_H_16_O_4_S.0.25H_2_O) C, H.

**General Procedure C: 4-(5-Bromothiophen-2-yl)-*N*-methylbenzamide (26).** Compound **25** (1.00 g, 3.53 mmol) was dissolved in THF (50 mL), then pyridine (2.79 g, 35.3 mmol) and pentafluorophenyltrifluoroacetate (4.95 g, 17.7 mmol) added. This mixture was stirred at room temperature for 2 h, then all solvents removed under reduced pressure to afford a yellow solid which was dissolved in EtOAc (150 mL), then washed with 1 M HCl (3x100 mL), water (100 mL) and brine (100 mL). Upon drying (Na_2_SO_4_) and filtration, the solvent was removed under reduced pressure and the resulting solid used directly in the next step. The pentafluorophenyl ester (794 mg, 1.77 mmol) was dissolved in THF (20 mL) and a 40% aqueous solution of methylamine (1.0 mL) added. After stirring for 2 h. at room temperature, all solvent was removed under reduced pressure and the resulting solid dissolved in CH_2_Cl_2_ (100 mL) and washed with sat. NaHCO_3_ (3x50 mL), water (50 mL) and brine (50 mL). The CH_2_Cl_2_ layer was dried (Na_2_SO_4_), filtered, and the solvent removed under reduced pressure. The crude product was purified by flash column chromatography on silica gel (5% acetone/CH_2_Cl_2_ as eluant), followed by trituration with Et_2_O to afford the title compound **26** as a pale yellow solid (398 mg, 76%), mp (CH_2_Cl_2_/Et_2_O) 203-205 ^o^C. ^1^H NMR [(CD_3_)_2_SO] δ 8.41-8.47 (br m, 1 H), 7.87 (d, *J* = 8.6 Hz, 2 H), 7.70 (d, *J* = 8.6 Hz, 2 H), 7.48 (d, *J* = 3.9 Hz, 1 H), 7.29 (d, *J* = 3.9 Hz, 1 H), 2.79 (d, *J* = 4.6 Hz, 3 H). LRMS (APCI^+^) calcd for C_12_H_11_BrNO_2_S 296, 298 (MH^+^), found 296, 298. Anal. (C_12_H_10_BrNOS) C, H, N

**General Procedure D: 4-(5-(4-Carboxy-3-(hydroxymethyl)phenyl)thiophen-2-yl)-2-hydroxybenzoic acid (44).** Compound **40** (155 mg, 0.41 mmol) was suspended in MeOH (15 mL), to which was added 2 M NaOH solution (15 mL). This mixture was heated at 100 ^o^C for 2 h. Upon cooling, the solution was partitioned between 1 M HCl (50 mL) and EtOAc (2x50 mL). The combined EtOAc fractions were dried (Na_2_SO_4_), filtered, and the solvent removed under reduced pressure to give the title compound **44** as a yellow-orange solid (150 mg, 100%). ^1^H NMR [(CD_3_)_2_SO] δ 12.90 (v br s, 2 H), 11.40 (v br s, 1 H), 8.03 (d, *J* = 1.7 Hz, 1 H), 7.92 (d, *J* = 8.2 Hz, 1 H), 7.83 (d, *J* = 8.7 Hz, 1 H), 7.76 (d, *J* = 3.9 Hz, 1 H), 7.72 (dd, *J* = 8.1, 2.0 Hz, 1 H), 7.69 (d, *J* = 3.9, 1 H), 7.28-7.32 (m, 2 H), 5.26 (v br s, 1 H), 4.89 (s, 2 H).

**General Procedure E: 2-Hydroxy-4-(5-(1-oxo-1,3-dihydroisobenzofuran-5-yl)thiophen-2-yl)benzoic acid (47).** Compound **44** (150 mg, 0.41 mmol) was suspended in a 1:1 mixture of TFA/CH_2_Cl_2_ (10 mL) and stirred at room temperature for 3 h. All solvent was rigorously removed under reduced pressure to give the title compound **47** (142 mg, 100%). ^1^H NMR [(CD_3_)_2_SO] δ 13.80 (v br s, 1 H), 11.40 (br s, 1 H), 8.00-8.06 (m, 1 H), 7.88-7.96 (m, 2 H), 7.83-7.87 (m, 1 H), 7.69-7.83 (m, 2 H), 7.27-7.33 (m, 2 H), 5.46 (s, 2 H). HRMS (EI^+^) calcd for C_19_H_12_O_5_S 352.0406 (M^+^), found 352.0411.

**General Procedure F: 2-Amino-4-(5-(1-oxo-1,3-dihydroisobenzofuran-5-yl)thiophen-2-yl)benzamide (55).** Compound **46** (50mg, 0.14 mmol) was dissolved in anhydrous DMF (3 mL), then 1-hydroxybenzotriazole (21 mg, 0.16 mmol) and EDCI (30 mg, 0.16 mmol) added. This mixture was stirred for 2 h. at room temperature then cooled to 0 ^o^C and c. NH_3_ added. The reaction was then allowed to warm back to room temperature and stirred for 1 h. The mixture was diluted with 5% NaHCO_3_ (30 mL) and extracted with EtOAc (2x30 mL). The combined EtOAc fractions were dried (Na_2_SO_4_), filtered and the solvent removed under reduced pressure to afford a yellow solid which was purified by flash column chromatography on silica gel (50% EtOAc/hexanes as eluant), followed by trituration with Et_2_O. The title compound **55** was isolated as a yellow solid (24 mg, 48%), mp (acetone) 282-286 ^o^C. ^1^H NMR [(CD_3_)_2_SO] δ 7.99 (s, 1 H), 7.86-7.95 (m, 2 H), 7.68-7.82 (m, 2 H), 7.62 (d, *J* = 8.3 Hz, 1 H), 7.57 (d, *J* = 3.9 Hz, 1 H), 7.08 (br s, 1 H), 7.02 (d, *J* = 1.6 Hz, 1 H), 6.88 (dd, *J* = 8.3, 1.6 Hz, 1 H), 6.71-6.78 (m, 2 H), 5.45 (s, 2 H). LRMS (APCI^-^) calcd for C_19_H_13_N_2_O_3_S 349 (M-H), found 349. Anal. (C_19_H_14_N_2_O_3_S) C, H, N.

**General Procedure G: 2-Hydroxy-*N*-(2-morpholinoethyl)-4-(5-(1-oxo-1,3-dihydroisobenzofuran-5-yl)thiophen-2-yl)benzamide (49).** Compound **47** (50 mg, 0.14 mmol) was suspended in pyridine (5 mL), to which was added 1-hydroxybenzotriazole (23 mg, 0.17 mmol), dicyclohexylcarbodiimide (35 mg, 0.17 mmol) and 4-(2-aminoethyl)morpholine (28 mg, 0.21 mmol). This mixture was stirred at 75 ^o^C for 5 h. The pyridine was removed under reduced pressure and the resulting residue dissolved in CH_2_Cl_2_ (50 mL). This solution was washed with water (2x50 mL), brine (50 mL) and dried (Na_2_SO_4_). Filtration and removal of the solvent under reduced pressure gave a crude solid which was purified by flash column chromatography on silica gel (1% MeOH/CH_2_Cl_2_ as eluant). The title compound **49** was isolated as a yellow solid (43 mg, 65%), mp (Et_2_O) 231-234 ^o^C. ^1^H NMR [(CD_3_)_2_SO] δ 12.71 (v br s, 1 H), 8.83 (br s, 1 H), 8.01 (s, 1 H), 7.88-7.96 (m, 3 H), 7.80 (d, *J* = 3.9 Hz, 1 H), 7.73 (d, *J* = 3.9 Hz, 1 H), 7.28 (dd, *J* = 8.2, 1.8 Hz, 1 H), 7.24 (d, *J* = 1.8 Hz, 1 H), 5.46 (s, 2 H), 3.58 (t, *J* = 4.6 Hz, 4 H), 3.45 (q, *J* = 6.3 Hz, 2 H), 2.50-2.55 (m, 2 H partly obscured), 2.43 (t, *J* = 4.4 Hz, 4 H). LRMS (APCI^+^) calcd for C_25_H_25_N_2_O_5_S 465 (MH^+^), found 465. Anal. (C_25_H_24_N_2_O_5_S.0.5H_2_O) C, H, N.

**5-(5-Phenylthiophen-2-yl)isobenzofuran-1(3*H*)-one (6).** Compound **5** was reacted with phenylboronic acid according to general procedure B. Purification was carried out by flash column chromatography on silica gel (50% EtOAc/hexanes) to give the title compound **6** as a yellow solid (66%), mp (EtOAc) 231-233 ^o^C. ^1^H NMR [(CD_3_)_2_SO] δ 7.99 (s, 1 H), 7.91 (dd, *J* = 1.4, 8.0 Hz, 1 H), 7.88 (d, *J* = 8.1 Hz, 1 H), 7.78 (d, *J* = 3.9 Hz, 1 H), 7.73 (d, *J* = 8.5 Hz, 2 H), 7.63 (d, *J* = 3.9 Hz, 1 H), 7.46 (t, *J* = 7.4 Hz, 2 H), 7.36 (tt, *J* = 1.1, 7.4 Hz, 1 H), 5.45 (s, 2 H). Anal. (C_18_H_12_O_2_S) C, H.

**5-(5-*o*-Tolylthiophen-2-yl)isobenzofuran-1(3*H*)-one (7).** Compound **5** was reacted with 2-tolylboronic acid according to general procedure B. The crude product was purified by flash column chromatography on silica gel (CH_2_Cl_2_ as eluant) to give the title compound **7** as a waxy yellow solid (59%), mp (Et_2_O) 109-111 ^o^C. ^1^H NMR [(CD_3_)_2_SO] δ 7.99 (br s, 1 H), 7.93 (dd, *J* = 8.1, 1.5 Hz, 1 H), 7.88 (d, *J* = 8.1, 1 H), 7.78 (d, *J* = 3.8 Hz, 1 H), 7.44-7.50 (m, 1 H), 7.27-7.38 (m, 3 H), 5.45 (s, 2 H), 2.46 (s, 3 H). LRMS (APCI^+^) calcd for C_19_H_15_O_2_S 307 (MH^+^), found 307. Anal. (C_19_H_14_O_2_S.0.25H_2_O) C, H.

**5-(5-*m*-Tolylthiophen-2-yl)isobenzofuran-1(3*H*)-one (8).** Compound **5** was reacted with 3-tolylboronic acid according to general procedure B. The crude product was purified by flash column chromatography on silica gel (CH_2_Cl_2_ as eluant) to give the title compound **8** as a yellow solid (63%), mp (Et_2_O) 192-194 ^o^C. ^1^H NMR [(CD_3_)_2_SO] δ 7.98 (br s, 1 H), 7.92 (dd, *J* = 8.1, 1.5 Hz, 1 H), 7.87 (d, *J* = 8.0 Hz, 1 H), 7.77 (d, *J* = 3.9 Hz, 1 H), 7.60 (d, *J* = 3.9 Hz, 1 H), 7.50-7.57 (m, 2 H), 7.35 (t, *J* = 7.6 Hz, 1 H), 7.18 (br d, *J* = 7.5 Hz, 1 H), 5.45 (s, 2 H), 2.37 (s, 3 H). LRMS (APCI^+^) calcd for C_19_H_15_O_2_S 307 (MH^+^), found 307. Anal. (C_19_H_14_O_2_S.0.25H_2_O) C, H

**5-(5-*p*-Tolylthiophen-2-yl)isobenzofuran-1(3*H*)-one (9).** Compound **5** was reacted with 4-tolylboronic acid according to general procedure B. The crude product was purified by flash column chromatography on silica gel (CH_2_Cl_2_ as eluant) to give the title compound **9** as a yellow solid (58%), mp (Et_2_O) 184-186 ^o^C. ^1^H NMR [(CD_3_)_2_SO] δ 7.97 (s, 1 H), 7.91 (dd, *J* = 8.1, 1.5 Hz, 1 H), 7.88 (d, *J* = 8.0 Hz, 1 H), 7.75 (d, *J* = 3.9 Hz, 1 H), 7.62 (d, *J* = 8.2 Hz, 2 H), 7.56 (d, *J* = 3.9 Hz, 1 H), 7.27 (d, *J* = 7.9 Hz, 2 H), 5.45 (s, 2 H), 2.34 (s, 3 H). LRMS (APCI^+^) calcd for C_19_H_15_O_2_S 307 (MH^+^), found 307. Anal. (C_19_H_14_O_2_S.0.25H_2_O) C, H.

**5-(5-(2-Chlorophenyl)thiophen-2-yl)isobenzofuran-1(3*H*)-one (10).** Compound **5** was reacted with 2-chlorophenylboronic acid according to general procedure B. The crude product was purified by flash column chromatography on silica gel (CH_2_Cl_2_ as eluant), followed by trituration with CH_2_Cl_2_/Et_2_O to give the title compound **10** as a yellow solid (89%), mp (CH_2_Cl_2_/Et_2_O) 131-133 ^o^C. ^1^H NMR [(CD_3_)_2_SO] δ 8.01 (s, 1 H), 7.94 (dd, *J* = 8.1, 1.5 Hz, 1 H), 7.89 (d, *J* = 7.9 Hz, 1 H), 7.80 (d, *J* = 3.9 Hz, 1 H), 7.74 (dd, *J* = 7.4, 1.9 Hz, 1 H), 7.62 (dd, *J* = 7.5, 2.0 Hz, 1 H), 7.55 (d, *J* = 3.9 Hz, 1 H), 7.40-7.49 (m, 2 H), 5.45 (s, 2 H). LRMS (APCI^+^) calcd for C_18_H_12_ClO_2_S 327 (MH^+^), found 327. Anal. (C_18_H_11_ClO_2_S.0.25H_2_O) C, H.

**5-(5-(3-Chlorophenyl)thiophen-2-yl)isobenzofuran-1(3*H*)-one (11).** Compound **5** was reacted with 3-chlorophenylboronic acid according to general procedure B. The crude product was purified by flash column chromatography on silica gel (CH_2_Cl_2_ as eluant) to give the title compound **11** as a yellow solid (77%), mp (Et_2_O) 207-209 ^o^C. ^1^H NMR [(CD_3_)_2_SO] δ 8.00 (br s, 1 H), 7.93 (dd, *J* = 8.1, 1.4 Hz, 1 H), 7.89 (d, *J* = 7.9 Hz, 1 H), 7.82 (t, *J* = 1.9 Hz, 1 H), 7.80 (d, *J* = 3.9 Hz, 1 H), 7.74 (d, *J* = 3.9 Hz, 1 H), 7.68 (ddd, *J* = 7.7, 1.6, 1.1 Hz, 1 H), 7.49 (t, *J* = 7.9 Hz, 1 H), 7.42 (ddd, *J* = 8.0, 2.0, 1.0 Hz, 1 H), 5.46 (s, 2 H). LRMS (APCI^+^) calcd for C_18_H_12_ClO_2_S 327 (MH^+^), found 327. Anal. (C_18_H_11_ClO_2_S.0.25H_2_O) C, H.

**5-(5-(4-Chlorophenyl)thiophen-2-yl)isobenzofuran-1(3*H*)-one (12).** Compound **5** was reacted with 4-chlorobenzeneboronic acid according to general procedure B. The crude product was purified by flash column chromatography on silica gel (CH_2_Cl_2_ as eluant), followed by trituration with CH_2_Cl_2_/Et_2_O to give the title compound **12** as a pale yellow solid (62%), mp (CH_2_Cl_2_/Et_2_O) 231-234 ^o^C. ^1^H NMR [(CD_3_)_2_SO] δ 7.99 (s, 1 H), 7.92 (dd, *J* = 8.1, 1.5 Hz, 1 H), 7.89 (d, *J* = 8.1 Hz, 1 H), 7.79 (d, *J* = 3.9 Hz, 1 H), 7.75 (d, *J* = 8.6 Hz, 2 H), 7.66 (d, *J* = 3.9 Hz, 1 H), 7.52 (d, *J* = 8.6 Hz, 2 H), 5.45 (s, 2 H). LRMS (APCI^+^) calcd for C_18_H_12_ClO_2_S 327 (MH^+^), found 327. Anal. (C_18_H_11_ClO_2_S) C, H

**5-(5-(2-Methoxyphenyl)thiophen-2-yl)isobenzofuran-1(3*H*)-one (13).** Compound **5** was reacted with 2-methoxyphenylboronic acid according to general procedure B. The crude product was purified by flash column chromatography on silica gel (CH_2_Cl_2_ as eluant), followed by trituration with Et_2_O to give the title compound **13** as a crystalline yellow solid (66%), mp (Et_2_O) 190-192 ^o^C. ^1^H NMR [(CD_3_)_2_SO] δ 7.98 (s, 1 H), 7.92 (dd, *J* = 8.1, 1.5 Hz, 1 H), 7.86 (d, *J* = 8.1 Hz, 1 H), 7.82 (dd, *J* = 7.8, 1.6 Hz, 1 H), 7.74 (d, *J* = 4.0 Hz, 1 H), 7.69 (d, *J* = 4.0 Hz, 1 H), 7.35 (ddd, *J* = 8.5, 7.4, 1.7 Hz, 1 H), 7.19 (dd, *J* = 8.3, 0.8 Hz, 1 H), 7.06 (ddd, *J* = 8.5, 7.7, 1.1 Hz, 1 H), 5.45 (s, 2 H), 3.96 (s, 3 H). LRMS (APCI^+^) calcd for C_19_H_15_O_3_S 323 (MH^+^), found 323. Anal. (C_19_H_14_O_3_S.0.5H_2_O) C, H.

**5-(5-(3-Methoxyphenyl)thiophen-2-yl)isobenzofuran-1(3*H*)-one (14).** Compound **5** was reacted with 3-methyoxyphenylboronic acid according to general procedure B. The crude product was purified by flash column chromatography on silica gel (CH_2_Cl_2_ as eluant), followed by trituration with Et_2_O to give the title compound **14** as a yellow solid (58%), mp (Et_2_O) 165-167 ^o^C. ^1^H NMR [(CD_3_)_2_SO] δ 7.99 (s, 1 H), 7.93 (dd, *J* = 8.1, 1.5 Hz, 1 H), 7.87 (d, *J* = 7.9 Hz, 1 H), 7.77 (d, *J* = 3.9 Hz, 1 H), 7.65 (d, *J* = 3.9 Hz, 1 H), 7.38 (t, *J* = 7.9 Hz, 1 H), 7.29 (ddd, *J* = 7.6, 1.5, 1.1 Hz, 1 H), 7.25 (t, *J* = 2.0 Hz, 1 H), 6.94 (ddd, *J* = 8.2, 2.5, 0.9 Hz, 1 H), 5.45 (s, 2 H), 3.83 (s, 3 H). LRMS (APCI^+^) calcd for C_19_H_15_O_3_S 323 (MH^+^), found 323. Anal. (C_19_H_14_O_3_S) C, H.

**5-(5-(4-Methoxyphenyl)thiophen-2-yl)isobenzofuran-1(3*H*)-one (15).** Compound **5** was reacted with 4-methyoxyphenylboronic acid according to general procedure B. The crude product was purified by flash column chromatography on silica gel (CH_2_Cl_2_ as eluant), followed by trituration with CH_2_Cl_2_/Et_2_O to give the title compound **15** as a yellow solid (53%), mp (CH_2_Cl_2_/Et_2_O) 224-227 ^o^C. ^1^H NMR [(CD_3_)_2_SO] δ 7.95 (s, 1 H), 7.90 (dd, *J* = 8.1, 1.5 Hz, 1 H), 7.86 (d, *J* = 8.1 Hz, 1 H), 7.73 (d, *J* = 3.9 Hz, 1 H), 7.66 (d, *J* = 8.9 Hz, 2 H), 7.49 (d, *J* = 3.9 Hz, 1 H), 7.03 (d, *J* = 8.9 Hz, 2 H), 5.44 (s, 2 H), 3.81 (s, 3 H). LRMS (APCI^+^) calcd for C_19_H_15_O_3_S 323 (MH^+^), found 323. Anal. (C_19_H_14_O_3_S.0.25H_2_O) C, H.

**5-(5-(3-Aminophenyl)thiophen-2-yl)isobenzofuran-1(3*H*)-one (16).** Compound **5** was reacted with 3-aminophenylboronic acid according to general procedure B. The desired product precipitated out of the reaction mixture, was collected by filtration and washed well with water and 10% MeOH/CH_2_Cl_2_ to give the title compound **16** as a dark yellow solid (56%), mp (CH_2_Cl_2_/Et_2_O) >300 ^o^C. ^1^H NMR [(CD_3_)_2_SO] δ 7.96 (s, 1 H), 7.90 (d, *J* = 8.0 Hz, 1 H), 7.88 (d, *J* = 7.9 Hz, 1 H), 7.72 (d, *J* = 3.8 Hz, 1 H), 7.43 (d, *J* = 3.8 Hz, 1 H), 7.08 (t, *J* = 7.7 Hz, 1 H), 6.86-3.89 (m, 2 H), 6.56 (dd, *J* = 1.3, 7.9 Hz, 1 H), 5.44 (s, 2 H), 5.23 (s, 2 H). HRMS (EI^+^) calcd for C_18_H_13_NO_2_S 307.0667 (M^+^), found 307.0669.

**5-(5-(4-Aminophenyl)thiophen-2-yl)isobenzofuran-1(3*H*)-one (17).** Compound **5** was reacted with 4-(4,4,5,5,-tetramethyl-1,3,2-dioxaboran-2-yl)aniline according to general procedure B. The crude product was purified by flash column chromatography on silica gel (5% acetone/CH_2_Cl_2_ as eluant), followed by trituration with Et_2_O to give the title compound **17** as a yellow solid (10%). ^1^H NMR [(CD_3_)_2_SO] δ 7.90 (s, 1 H), 7.81-7.87 (m, 2 H), 7.67 (d, *J* = 3.9 Hz, 1 H), 7.39 (d, *J* = 8.5 Hz, 2 H), 7.30 (d, *J* = 3.9 Hz, 1 H), 6.62 (d, *J* = 8.6 Hz, 2 H), 5.43 (br s, 4 H). HRMS (EI^+^) calcd for C_18_H_13_NO_2_S 307.0667 (MH^+^), found 307.0673.

**5-(5-(3-Hydroxyphenyl)thiophen-2-yl)isobenzofuran-1(3*H*)-one (18).** Compound **5** was reacted with 3-hydroxyphenylboronic acid according to general procedure B. The crude product was purified by flash column chromatography on silica gel (10% acetone/CH_2_Cl_2_ as eluant), followed by trituration with CH_2_Cl_2_/Et_2_O to give the title compound **18** as a yellow solid (28%), mp (CH_2_Cl_2_/Et_2_O) 276-279 ^o^C. ^1^H NMR [(CD_3_)_2_SO] δ 9.64 (s, 1 H), 7.98 (s, 1 H), 7.91 (dd, *J* = 1.4, 8.1 Hz, 1 H), 7.86 (d, *J* = 8.0 Hz, 1 H), 7.74 (d, *J* = 3.8 Hz, 1 H), 7.53 (d, *J* = 3.8 Hz, 1 H), 7.25 (t, *J* = 739 Hz, 1 H), 7.15 (d, *J* = 8.1 Hz, 1 H), 7.09 (t, *J* = 1.95 Hz, 1 H), 6.77 (ddd, *J* = 0.8, 2.3, 8.0 Hz, 1 H), 5.45 (s, 2 H). LRMS (APCI^+^) calcd for C_18_H_13_O_3_S 309 (MH^+^), found 309. Anal. (C_18_H_12_O_3_S.0.5H_2_O) C, H.

**5-(5-(4-Hydroxyphenyl)thiophen-2-yl)isobenzofuran-1(3*H*)-one (19).** Compound **5** was reacted with 4-hydroxyphenylboronic acid according to general procedure B. The crude product was purified by flash column chromatography on silica gel (10% acetone/CH_2_Cl_2_ as eluant), followed by trituration with CH_2_Cl_2_/Et_2_O to give the title compound **19** as a yellow solid (62%), mp (CH_2_Cl_2_/Et_2_O) 279-282 ^o^C. ^1^H NMR [(CD_3_)_2_SO] δ 9.71 (s, 1 H), 7.94 (s, 1 H), 7.82-7.90 (m, 2 H), 7.71 (d, *J* = 3.9 Hz, 1 H), 7.54 (d, *J* = 8.7 Hz, 2 H), 7.41 (d, *J* = 3.9 Hz, 1 H), 6.84 (d, *J* = 8.7 Hz, 2 H), 5.43 (s, 2 H). Anal. (C_18_H_12_O_3_S.0.25H_2_O) C, H.

**3-(5-(1-Oxo-1,3-dihydroisobenzofuran-5-yl)thiophen-2-yl)benzonitrile (20).** Compound **5** was reacted with 3-cyanobenzeneboronic acid according to general procedure B. The crude product was purified by flash column chromatography on silica gel (CH_2_Cl_2_ as eluant), followed by trituration with CH_2_Cl_2_/Et_2_O to give the title compound **20** as a yellow solid (58%), mp (CH_2_Cl_2_/Et_2_O) 250-251 ^o^C. ^1^H NMR [(CD_3_)_2_SO] δ 8.24 (t, *J* = 1.5 Hz, 1 H), 8.03 (ddd, *J* = 8.0, 1.8, 1.1 Hz, 1 H), 8.01 (br s, 1 H), 7.94 (dd, *J* = 8.1, 1.4 Hz, 1 H), 7.90 (d, *J* = 8.0 Hz, 1 H), 7.78-7.84 (m, 3 H), 7.67 (t, *J* = 7.8 Hz, 1 H), 5.46 (s, 2 H). LRMS (APCI^+^) calcd for C_19_H_12_NO_2_S 318 (MH^+^), found 318. Anal. (C_19_H_11_NO_2_S.0.25H_2_O) C, H, N.

**4-(5-(1-Oxo-1,3-dihydroisobenzofuran-5-yl)thiophen-2-yl)benzonitrile (21).** Compound **5** was reacted with 4-cyanobenzeneboronic acid according to general procedure B. The crude product was purified by flash column chromatography on silica gel (CH_2_Cl_2_ as eluant), followed by trituration with CH_2_Cl_2_/Et_2_O to give the title compound **21** as a yellow solid (76%), mp (CH_2_Cl_2_/Et_2_O) >300 ^o^C. ^1^H NMR [(CD_3_)_2_SO] δ 8.02 (br s, 1 H), 7.88-7.96 (m, 6 H), 7.82-7.86 (m, 2 H), 5.46 (s, 2 H). LRMS (APCI^+^) calcd for C_19_H_12_NO_2_S 318 (MH^+^), found 318. Anal. (C_19_H_11_NO_2_S.0.25H_2_O) H, N. C; +0.5.

**3-(5-(1-Oxo-1,3-dihydroisobenzofuran-5-yl)thiophen-2-yl)benzamide (22).** Compound **5** was reacted with 3-aminocarbonylphenylboronic acid according to general procedure B. The desired product precipitated out of the reaction mixture, was collected by filtration and washed well with water and 10% MeOH/CH_2_Cl_2_ to give the title compound **22** as an olive green solid (73%), mp (CH_2_Cl_2_/Et_2_O) 259-263 ^o^C. ^1^H NMR [(CD_3_)_2_SO] δ 8.21 (t, *J* = 1.6 Hz, 1 H), 8.09 (s, 1 H), 8.01 (s, 1 H), 7.94 (dd, *J* = 1.4, 8.1 Hz, 1 H), 7.82-7.91 (m, 3 H), 7.81 (d, *J* = 3.9 Hz, 1 H), 7.71 (d, *J* = 3.9 Hz, 1 H), 7.54 (t, *J* = 7.8 Hz, 1 H), 7.45 (s, 1 H), 5.45 (s, 2 H). LRMS (APCI^+^) calcd for C_19_H_14_NO_3_S 336 (MH^+^), found 336. Anal. (C_19_H_13_NO_3_S.0.5H_2_O) C, H, N.

**4-(5-(1-Oxo-1,3-dihydroisobenzofuran-5-yl)thiophen-2-yl)benzamide (23).** Compound **5** was reacted with 4-aminocarbonylphenylboronic acid according to general procedure B. The desired product precipitated out of the reaction mixture, was collected by filtration and washed well with water and 10% MeOH/CH_2_Cl_2_ to give the title compound **23** as a green-yellow solid (44%), mp (CH_2_Cl_2_/MeOH) 297-300 ^o^C. ^1^H NMR [(CD_3_)_2_SO] δ 7.92-8.03 (m, 5 H), 7.89 (d, *J* = 8.0 Hz, 1 H), 7.79-7.84 (m, 3 H), 7.75 (d, *J* = 3.9 Hz, 1 H), 7.37 (s, 1 H), 5.46 (s, 2 H). LRMS (APCI^+^) calcd for C_19_H_14_NO_3_S 336 (MH^+^), found 336. Anal. (C_19_H_13_NO_3_S.0.75H_2_O) C, H, N.

**4-(5-(1-Oxoisoindolin-5-yl)thiophen-2-yl)benzamide (23a)**. 5-(5-Bromothiophen-2-yl)isoindolin-1-one^16^ was reacted with 4-aminocarbonylphenylboronic acid according to general procedure B. The desired product precipitated out of the reaction mixture, was collected by filtration and washed well with water and 10% MeOH/CH_2_Cl_2_. This solid was dissolved in a small quantity of warm DMSO, then drops of water added until precipitation began. Upon cooling the solid was collected by filtration, washed well with water and dried to give the title compound **23a** as a pale green solid (18%), mp (DMSO/water) >295 ^o^C. ^1^H NMR [400 MHz, (CD_3_)_2_SO] δ 8.54 (br s, 1 H), 7.90-8.03 (m, 4 H), 7.76-7.85 (m, 3 H), 7.69-7.74 (m, 3 H), 7.36 (br s, 1 H), 4.43 (s, 2 H). LRMS (APCI^+^) calcd for C_19_H_15_N_2_O_2_S 335 (MH^+^), found 335. Anal. (C_19_H_14_N_2_O_2_S.0.75H_2_O) C, H, N.

**4-(5-(2-methyl-1-oxoisoindolin-5-yl)thiophen-2-yl)benzamide (23b)**. 5-(5-Bromothiophen-2-yl)-2-methylisoindolin-1-one^16^ was reacted with 4-aminocarbonylphenylboronic acid according to general procedure B. Upon cooling, the mixture was partitioned between CH_2_Cl_2_ (50 mL) and water (50 mL). The solid which precipitated from this biphasic mixture was collected by filtration, washed well with water and dried under vacuum. This crude product was purified by by flash column chromatography on silica gel (5% MeOH/CH_2_Cl_2_ as eluant) to give the title compound **23b** as a pale yellow crystalline solid (70%), mp (CH_2_Cl_2_) 287-290 ^o^C. ^1^H NMR [(CD_3_)_2_SO] δ 8.00 (br s, 1 H), 7.92-7.96 (m, 4 H), 7.77-7.84 (m, 3 H), 7.69-7.73 (m, 3 H), 7.36 (br s, 1 H), 4.52 (s, 2 H), 3.09 (s, 3 H). LRMS (APCI^+^) calcd for C_20_H_17_N_2_O_2_S 348 (MH^+^), found 348. Anal. (C_20_H_16_N_2_O_2_S.0.25H_2_O) C, H, N.

**4-(5-Bromothiophen-2-yl)-*N,N*-dimethylbenzamide (27).** Compound **25** was reacted with pentafluorophenyltrifluoroacetate, followed by a 40% aqueous solution of methylamine, according to general procedure C. The crude product was purified by trituration with Et_2_O to give the title compound **27** as a yellow solid (73%). ^1^H NMR in agreement with literature values.^17^

***N*-Methyl-4-(5-(1-oxo-1,3-dihydroisobenzofuran-5-yl)thiophen-2-yl)benzamide (29).** Compounds **26** and **28** were reacted according to general procedure B. The crude product was purified by flash column chromatography on silica gel (5% acetone/CH_2_Cl_2_ as eluant), followed by trituration with Et_2_O/CH_2_Cl_2_ to give the title compound **29** as a yellow solid (75%), mp (CH_2_Cl_2_/Et_2_O) 299-302 ^o^C. ^1^H NMR [(CD_3_)_2_SO] δ 8.43-8.49 (br m, 1 H), 8.01 (s, 1 H), 7.87-7.89 (m, 4 H), 7.79-7.84 (m, 3 H), 7.74 (d, *J* = 3.9 Hz, 1 H), 5.46 (s, 2 H), 2.80 (d, *J* = 4.5 Hz, 3 H). LRMS (APCI^+^) calcd for C_20_H_16_NO_3_S 350 (MH^+^), found 350. Anal. (C_20_H_15_NO_3_S.0.5H_2_O) C, H, N.

***N,N*-Dimethyl-4-(5-(1-oxo-1,3-dihydroisobenzofuran-5-yl)thiophen-2-yl)benzamide (30).** Compounds **27** and **28** were reacted according to general procedure B. The crude product was purified by flash column chromatography on silica gel (5% acetone/CH_2_Cl_2_ as eluant), followed by trituration with Et_2_O/CH_2_Cl_2_ to give the title compound **30** as a yellow solid (57%), mp (CH_2_Cl_2_/Et_2_O) 290-292 ^o^C. ^1^H NMR [(CD_3_)_2_SO] δ 8.00 (s, 1 H), 7.94 (dd, *J* = 8.1, 1.5 Hz, 1 H), 7.89 (d, *J* = 8.1 Hz, 1 H), 7.81 (d, *J* = 3.9 Hz, 1 H), 7.78 (d, *J* = 8.4 Hz, 2 H), 7.71 (d, *J* = 3.9 Hz, 1 H), 7.49 (d, *J* = 8.4 Hz, 2 H), 5.46 (s, 2 H), 2.98 (br s, 6 H). LRMS (APCI^+^) calcd for C_21_H_18_NO_3_S 364 (MH^+^), found 364. Anal. (C_21_H_17_NO_3_S.0.25H_2_O) C, H, N.

**5-(5-(4-(4-Methylpiperazine-1-carbonyl)phenyl)thiophen-2-yl)isobenzofuran-1(3*H*)-one (31).** Compound **5** was reacted with 4-(4-methylpiperazine-1-carbonyl)phenylboronic acid pinacol ester according to general procedure B. The crude product was purified by flash column chromatography on silica gel (5% MeOH/CH_2_Cl_2_ as eluant), followed by trituration with CH_2_Cl_2_/Et_2_O to give the title compound **31** as a pale yellow-green solid (61%), mp (CH_2_Cl_2_/Et_2_O) >300 ^o^C. ^1^H NMR [(CD_3_)_2_SO] δ 8.00 (s, 1 H), 7.94 (dd, *J* = 1.4, 8.1 Hz, 1 H), 7.89 (d, *J* = 8.0 Hz, 1 H), 7.81 (d, *J* = 3.9 Hz, 1 H), 7.79 (d, *J* = 8.3 Hz, 2 H), 7.70 (d, *J* = 3.9 Hz, 1 H), 7.46 (d, *J* = 8.3 Hz, 2 H), 5.45 (s, 2 H), 3.51 (s, 4 H), 2.32 (s, 4 H), 2.20 (s, 3 H). LRMS (APCI^+^) calcd for C_24_H_23_N_2_O_3_S 419 (MH^+^), found 419. Anal. (C_24_H_22_N_2_O_3_S) C, H, N.

***N*-(3-(Dimethylamino)propyl)-4-(5-(1-oxo-1,3-dihydroisobenzofuran-5-yl)thiophen-2-yl)benzamide (32).** Compound **5** was reacted with *N*-[3-(*N,N’*-dimethylamino)propyl]benzamide-4-phenylboronic acid pinacol ester according to general procedure B. The desired product precipitated out of the reaction mixture, was collected by filtration and washed well with water and 10% MeOH/CH_2_Cl_2_, then triturated with CH_2_Cl_2_/Et_2_O to give the title compound **32** as a yellow solid (42%), mp (CH_2_Cl_2_/Et_2_O) 185-188 ^o^C. ^1^H NMR [(CD_3_)_2_SO] δ 8.53 (t, *J* = 5.4 Hz, 1 H), 8.01 (s, 1 H), 7.87-7.96 (m, 4 H), 7.79-7.84 (m, 3 H), 7.74 (d, *J* = 3.9 Hz, 1 H), 5.46 (s, 2 H), 3.32 (m, 2 H), 2.28 (t, *J* = 7.1 Hz, 2 H), 2.15 (s, 6 H), 1.67 (q, *J* = 7.1 Hz, 2 H). LRMS (APCI^+^) calcd for C_24_H_25_N_2_O_3_S 421 (MH^+^), found 421. Anal. (C_24_H_24_N_2_O_3_S.0.75H_2_O) C, H, N.

**Ethyl 2-amino-4-(5-bromothiophen-2-yl)benzoate (37).** Compound **34** was reacted with 2-bromothiophene according to general procedure A. The crude product was purified by flash column chromatography on silica gel (5% EtOAc/hexanes as eluant) to afford the title compound **37** as a waxy yellow solid (46%). ^1^H NMR [(CD_3_)_2_SO] δ 7.72 (d, *J* = 8.4 Hz, 1 H), 7.35 (d, *J* = 3.9 Hz, 1 H), 7.28 (d, *J* = 3.9 Hz, 1 H), 6.99 (d, *J* = 1.8 Hz, 1 H), 6.82 (dd, *J* = 8.4, 1.9 Hz, 1 H), 6.73 (br s, 2 H), 4.26 (q, *J* = 7.1 Hz, 2 H), 1.30 (t, *J* = 7.1 Hz, 3 H). HRMS (EI^+^) calcd for C_13_H_12_^79^BrNO_2_S 324.9772 (M^+^), found 324.9772; calcd for C_13_H_12_^81^BrNO_2_S 326.9752 (M^+^), found 326.9756.

**Ethyl 4-(5-bromothiophen-2-yl)-2-hydroxybenzoate (38).** Compound **35** and 2-bromothiophene were reacted according to general procedure A. The crude product was purified by flash column chromatography on silica gel (10% EtOAc/hexanes as eluant), followed by trituration with 10% Et_2_O/hexanes to give the title compound **38** as a pale yellow solid (68%), mp (Et_2_O/hexanes) 92-94 ^o^C. ^1^H NMR [(CD_3_)_2_SO] δ 10.67 (br s, 1 H), 7.81 (d, *J* = 8.2 Hz, 1 H), 7.54 (d, *J* = 4.0 Hz, 1 H), 7.31 (d, *J* = 4.0 Hz, 1 H), 7.18-7.24 (m, 2 H), 4.37 (q, *J* = 7.1 Hz, 2 H), 1.35 (t, *J* = 7.1 Hz, 3 H). LRMS (APCI^-^) calcd for C_13_H_10_BrO_3_S 325, 327 (M-H), found 325, 327. Anal. (C_13_H_11_BrO_3_S) C, H.

**Ethyl 2-hydroxy-4-(5-(1-oxo-1,3-dihydroisobenzofuran-5-yl)thiophen-2-yl)benzoate (40).** Compounds **38** and **28** were reacted according to general procedure B. The crude product was purified by flash column chromatography on silica gel (10% acetone/CH_2_Cl_2_ as eluant) to give the title compound **40** as a yellow-orange solid (76%), mp (Et_2_O) 208-210 ^o^C. ^1^H NMR [(CD_3_)_2_SO] δ 10.70 (s, 1 H), 7.92-8.06 (m, 3 H), 7.89 (d, *J* = 8.0 Hz, 1 H), 7.85 (d, *J* = 8.1 Hz, 1 H), 7.81 (dd, *J* = 9.2, 4.0 Hz, 1 H), 7.30-7.35 (m, 2 H), 5.46 (s, 2 H), 4.38 (q, *J* = 7.1 Hz, 2 H), 1.36 (t, *J* = 7.1 Hz, 3 H). LRMS (APCI^+^) calcd for C_21_H_17_O_5_S 381 (MH^+^), found 381. Anal. (C_21_H_16_O_5_S) C, H.

**2-Amino-4-(5-(4-carboxy-3-(hydroxymethyl)phenyl)thiophen-2-yl)benzoic acid (43).** Compound **37** was reacted with **28** according to general procedure B. This product was contaminated with ca 20% of dimer resulting from homo-coupling of the bromide, thus was deprotected directly to the acid according to general procedure D. The undesired dimer was then separated by filtration of the reaction mixture prior to acidification to isolate the desired product. The title compound **43** was isolated as a yellow solid (48% over 2 steps). ^1^H NMR [(CD_3_)_2_SO] δ 8.02 (d, *J* = 1.4 Hz, 1 H), 7.92 (d, *J* = 8.2 Hz, 1 H), 7.75 (d, *J* = 8.4 Hz, 1 H), 7.71 (dd, *J* = 8.1, 1.9 Hz, 1 H), 7.67 (d, *J* = 3.9 Hz, 1 H), 7.56 (d, *J* = 3.9 Hz, 1 H), 7.11 (d, *J* = 1.8 Hz, 1 H), 6.92 (dd, *J* = 8.4, 1.8 Hz, 1 H), 5.33 (v br s, 1 H), 4.88 (s, 2 H). Note all remaining exchangeable protons too broad to integrate. LRMS (APCI^+^) calcd for C_19_H_16_NO_5_S 370 (MH^+^), found 370.

**2-Amino-4-(5-(1-oxo-1,3-dihydroisobenzofuran-5-yl)thiophen-2-yl)benzoic acid (46)**. Compound **43** was treated with TFA/CH_2_Cl_2_ according to general procedure E. The crude solid was triturated with 10% MeOH/CH_2_Cl_2_ to afford the title compound **46** as a yellow solid (43%), mp (CH_2_Cl_2_/MeOH) 263 ^o^C dec. ^1^H NMR [(CD_3_)_2_SO] δ 8.50 (v br s, 3 H), 7.99 (s, 1 H), 7.92 (d, *J* = 8.2 Hz, 1 H), 7.87 (d, *J* = 8.0 Hz, 1 H), 7.77 (d, *J* = 3.9 Hz, 1 H), 7.76 (d, *J* = 8.4 Hz, 1 H), 7.59 (d, *J* = 3.9 Hz, 1 H), 7.08 (d, *J* = 1.7 Hz, 1 H), 6.91 (dd, *J* = 8.4, 1.8 Hz, 1 H), 5.45 (s, 2 H). HRMS (FAB^+^) calcd for C_19_H_14_NO_4_S 352.0644 (MH^+^), found 352.0645.

**2-Hydroxy-4-(5-(1-oxo-1,3-dihydroisobenzofuran-5-yl)thiophen-2-yl)benzamide (48).** Compound **47** was reacted with pentafluorophenyltrifluoroacetate, followed by 0.5 M NH_3_ in dioxane according to general procedure C. The crude product was purified by trituration with 10% MeOH/CH_2_Cl_2_ to give the title compound **48** as a yellow solid (57%), mp (MeOH/CH_2_Cl_2_) 297-300 ^o^C dec. ^1^H NMR [(CD_3_)_2_SO] δ 13.22 (s, 1 H), 8.42 (br s, 1 H), 7.99-8.04 (m, 2 H), 7.87-7.95 (m, 3 H), 7.79 (d, *J* = 3.9 Hz, 1 H), 7.75 (d, *J* = 3.9 Hz, 1 H), 7.25 (dd, *J* = 8.3, 1.9 Hz, 1 H), 7.22 (d, *J* = 1.8 Hz, 1 H), 5.46 (s, 2 H). LRMS (APCI^+^) calcd for C_19_H_14_NO_4_S 352 (MH^+^), found 352. Anal. (C_19_H_13_NO_4_S.0.5H_2_O) C, H, N.

***N*-(2-(Dimethylamino)ethyl)-2-hydroxy-4-(5-(1-oxo-1,3-dihydroisobenzofuran-5-yl)thiophen-2-yl)benzamide (50).** Compound **47** was reacted with *N,N*-dimethylethylenediamine according to general procedure G. The resulting product was purified by recrystallisation from THF/MeOH, to give the title compound **50** as a yellow solid (57%), mp (THF/MeOH) 242-245 ^o^C. ^1^H NMR [(CD_3_)_2_SO] δ 8.85 (br t, *J* = 5.4 Hz, 1 H), 8.01 (s, 1 H), 7.87-7.96 (m, 3 H), 7.80 (d, *J* = 3.9 Hz, 1 H), 7.72 (d, *J* = 3.9 Hz, 1 H), 7.28 (dd, *J* = 8.2, 1 8 Hz, 1 H), 7.23 (d, *J* = 1.8 Hz, 1 H), 5.46 (s, 2 H), 3.46 (q, *J* = 6.1 Hz, 2 H), 2.63 (t, *J* = 6.5 Hz, 2 H), 2.34 (s, 6 H). OH too broad to observe. HRMS (FAB^+^) calcd for C_23_H_23_N_2_O_4_S 423.1379 (MH^+^), found 423.1381.

**2-Hydroxy-*N*-(2-hydroxyethyl)-4-(5-(1-oxo-1,3-dihydroisobenzofuran-5-yl)thiophen-2-yl)benzamide (51).** Compound **47** was reacted with 2-aminoethanol according to general procedure G. The resulting product was purified by flash column chromatography on silica gel (2% MeOH/CH_2_Cl_2_ as eluant), followed by recrystallisation from THF/MeOH, to give the title compound **51** as a yellow solid (29%), mp (THF/MeOH) 240-242 ^o^C. ^1^H NMR [(CD_3_)_2_SO] δ 12.80 (s, 1 H), 8.84 (t, *J* = 5.5 Hz, 1 H), 8.01 (s, 1 H), 7.92-7.98 (m, 2 H), 7.89 (d, *J* = 8.1 Hz, 1 H), 7.80 (d, *J* = 3.9 Hz, 1 H), 7.74 (d, *J* = 3.9 Hz, 1 H), 7.28 (dd, *J* = 8.3, 1.9 Hz, 1 H), 7.23 (d, *J* = 1.9 Hz, 1 H), 5.46 (s, 2 H), 4.78 (t, *J* = 5.5 Hz, 1 H), 3.56 (q, *J* = 5.8 Hz, 2 H), 3.39 (q, *J* = 5.8 Hz, 2 H). LRMS (APCI^+^) calcd for C_21_H_18_NO_5_S 396 (MH^+^), found 396. Anal. (C_21_H_17_NO_5_S.0.25H_2_O) C, H, N.

**2-Hydroxy-*N*-(2-hydroxypropyl)-4-(5-(1-oxo-1,3-dihydroisobenzofuran-5-yl)thiophen-2-yl)benzamide (52).** Compound **47** was reacted with 1-amino-2-propanol according to general procedure G. The resulting product was purified by recrystallisation from THF/MeOH, to give the title compound **52** as a yellow solid (19%), mp (THF/MeOH) 241-244 ^o^C. ^1^H NMR [(CD_3_)_2_SO] δ 12.72 (s, 1 H), 8.81 (br s, 1 H), 8.01 (s, 1 H), 7.92-7.99 (m, 2 H), 7.89 (d, *J* = 8.0 Hz, 1 H), 7.80 (d, *J* = 4.0 Hz, 1 H), 7.73 (d, *J* = 3.9 Hz, 1 H), 7.28 (8.3, 1.6 Hz, 1 H), 7.23 (d, *J* = 1.7 Hz, 1 H), 5.46 (s, 2 H), 4.80 (d, *J* = 4.8 Hz, 1 H), 3.83 (pentet, *J* = 5.7 Hz, 2 H), 3.16-3.26 (m, 2 H), 1.09 (d, *J* = 6.2 Hz, 3 H). HRMS (FAB^+^) calcd for C_22_H_20_NO_5_S 410.1062 (MH^+^), found 410.1071.

***N*-(2-Amino-2-oxoethyl)-2-hydroxy-4-(5-(1-oxo-1,3-dihydroisobenzofuran-5-yl)thiophen-2-yl)benzamide (53).** Compound **47** was reacted with glycinamide hydrochloride according to general procedure G. The resulting product was purified by recrystallisation from THF/MeOH, to give the title compound **53** as a yellow solid (28%), mp (THF/MeOH) 270-274 ^o^C. ^1^H NMR [(CD_3_)_2_SO] δ 12.47 (s, 1 H), 9.05 (t, *J* = 5.5 Hz, 1 H), 8.01 (s, 1 H), 7.95 (d, *J* = 8.4 Hz, 1 H), 7.94 (dd, *J* = 8.1, 1.4 Hz, 1 H), 7.89 (d, *J* = 8.1 Hz, 1 H), 7.80 (d, *J* = 3.9 Hz, 1 H), 7.73 (d, *J* = 4.0 Hz, 1 H), 7.47 (br s, 1 H), 7.31 (dd, *J* = 8.3, 1.8 Hz, 1 H), 7.25 (d, *J* = 1.8 Hz, 1 H), 7.08 (br s, 1 H), 5.46 (s, 2 H), 3.89 (d, *J* = 5.6 Hz, 2 H). HRMS (FAB^+^) calcd for C_21_H_17_N_2_O_5_S 409.0858 (MH^+^), found 409.0850.

***N*-(3-Amino-3-oxopropyl)-2-hydroxy-4-(5-(1-oxo-1,3-dihydroisobenzofuran-5-yl)thiophen-2-yl)benzamide (54).** Compound **47** was reacted with 3-aminopropanamide according to general procedure G. The resulting product was purified by flash column chromatography on silica gel (1% MeOH/CH_2_Cl_2_ as eluant). The title compound **54** was isolated as a yellow solid (13%). ^1^H NMR [(CD_3_)_2_SO] δ 12.73 (s, 1 H), 8.90 (t, *J* = 5.5 Hz, 1 H), 8.01 (s, 1 H), 7.87-7.96 (m, 3 H), 7.80 (d, *J* = 3.9 Hz, 1 H), 7.73 (d, *J* = 3.9 Hz, 1 H), 7.36 (br s, 1 H), 7.27 (dd, *J* = 8.3, 1.8 Hz, 1 H), 7.22 (d, *J* = 1.8 Hz, 1 H), 6.83 (br s, 1 H), 5.46 (s, 2 H), 3.50 (q, *J* = 6.5 Hz, 2 H), 2.39 (t, *J* = 7.0 Hz, 2 H). HRMS (FAB^+^) calcd for C_22_H_19_N_2_O_5_S 423.1015 (MH^+^), found 423.1010.

**2-Amino-*N*-(2-morpholinoethyl)-4-(5-(1-oxo-1,3-dihydroisobenzofuran-5-yl)thiophen-2-yl)benzamide (56).** Compound **46** was reacted with 4-(2-aminoethyl)morpholine according to general procedure F. The resulting product was purified by column chromatography on neutral alumina (1% MeOH/CH_2_Cl_2_ as eluant), followed by trituration with Et_2_O, to give the title compound **56** as a yellow solid (80%), mp (Et_2_O) 209-211 ^o^C. ^1^H NMR [(CD_3_)_2_SO] δ 8.15 (t, J = 5.6 Hz, 1 H), 7.98 (s, 1 H), 7.92 (dd, *J* = 8.1, 1.4 Hz, 1 H), 7.88 (d, *J* = 8.0 Hz, 1 H), 7.77 (d, *J* = 3.9 Hz, 1 H), 7.56 (d, *J* = 3.9 Hz, 1 H), 7.53 (d, *J* = 8.3 Hz, 1 H), 7.03 (d, *J* = 1.9 Hz, 1 H), 6.92 (dd, *J* = 8.2, 1.9 Hz, 1 H), 6.56 (br s, 2 H), 5.45 (s, 2 H), 3.58 (t, *J* = 4.6 Hz, 4 H), 3.28-3.39 (m, 4 H), 2.36-2.47 (m, 4 H). LRMS (APCI^+^) calcd for C_25_H_26_N_3_O_4_S 464 (MH^+^), found 464. Anal. (C_25_H_25_N_3_O_4_S) C, H, N.

**2-Amino-*N*-(2-(dimethylamino)ethyl)-4-(5-(1-oxo-1,3-dihydroisobenzofuran-5-yl)thiophen-2-yl)benzamide (57).** Compound **46** was reacted with *N,N*-dimethylethylenediamine according to general procedure F. The resulting product was purified by column chromatography on neutral alumina (2% MeOH/CH_2_Cl_2_ as eluant), followed by trituration with Et_2_O, to give the title compound **57** as a yellow solid (38%), mp (Et_2_O) 185-188 ^o^C. ^1^H NMR [(CD_3_)_2_SO] δ 8.13 (t, *J* = 5.6 Hz, 1 H), 7.98 (s, 1 H), 7.92 (dd, *J* = 8.1, 1.3 Hz, 1 H), 7.86 (d, *J* = 7.9 Hz, 1 H), 7.76 (d, *J* = 3.9 Hz, 1 H), 7.56 (d, *J* = 3.9 Hz, 1 H), 7.53 (d, *J* = 8.3 Hz, 1 H), 7.03 (d, *J* = 1.9 Hz, 1 H), 6.90 (dd, *J* = 8.2, 1.8 Hz, 1 H), 6.58 (br s, 2 H), 5.45 (s, 2 H), 3.25-3.35 (m, 2 H; partly obscured by H_2_O), 2.40 (t, *J* = 6.9 Hz, 2 H), 2.19 (s, 6 H).Hz, 1 H) HRMS (FAB^+^) calcd for C_23_H_24_N_3_O_3_S 422.1538 (MH^+^), found 422.1541.

**5-(5-(4-(Methylthio)phenyl)thiophen-2-yl)isobenzofuran-1(3*H*)-one (58).** Compound **5** was reacted with 4-(methylthio)benzeneboronic acid according to general procedure B. The crude product was purified by flash column chromatography on silica gel (CH_2_Cl_2_ as eluant), followed by trituration with CH_2_Cl_2_/Et_2_O to give the title compound **58** as a bright yellow solid (64%), mp (CH_2_Cl_2_/Et_2_O) 239-241 ^o^C. ^1^H NMR [(CD_3_)_2_SO] δ 7.97 (br s, 1 H), 7.91 (dd, *J* = 8.1, 1.4 Hz, 1 H), 7.87 (d, *J* = 8.0 Hz, 1 H), 7.76 (d, *J* = 3.9 Hz, 1 H), 7.67 (d, *J* = 8.5 Hz, 2 H), 7.60 (d, *J* = 3.9 Hz, 1 H), 7.33 (d, *J* = 8.6 Hz, 1 H), 5.45 (s, 2 H), 2.52 (s, 3 H). LRMS (APCI^+^) calcd for C_19_H_15_O_2_S_2_ 339 (MH^+^), found 339. Anal. (C_19_H_14_O_2_S_2_.0.25H_2_O) C, H.

**5-(5-(4-(Hydroxymethyl)phenyl)thiophen-2-yl)isobenzofuran-1(3*H*)-one (59).** Compound **5** was reacted with 4-(hydroxymethyl)phenylboronic acid according to general procedure B. The crude product was purified by flash column chromatography on silica gel (CH_2_Cl_2_ as eluant), followed by trituration with CH_2_Cl_2_/Et_2_O to give the title compound **59** as a yellow solid (79%), mp (CH_2_Cl_2_/Et_2_O) 239-241 ^o^C. ^1^H NMR [(CD_3_)_2_SO] δ 7.98 (br s, 1 H), 7.92 (dd, *J* = 8.1, 1.5 Hz, 1 H), 7.87 (d, *J* = 8.0 Hz, 1 H), 7.76 (d, *J* = 3.9 Hz, 1 H), 7.69 (d, *J* = 8.3 Hz, 2 H), 7.60 (d, *J* = 3.9 Hz, 1 H), 7.40 (d, *J* = 8.4 Hz, 2 H), 5.45 (s, 2 H), 5.22 (t, *J* = 5.7 Hz, 1 H), 4.53 (d, *J* = 5.6 Hz, 2 H). LRMS (APCI^+^) calcd for C_19_H_15_O_3_S 323 (MH^+^), found 323. Anal. (C_19_H_14_O_3_S) C, H.

**5-(5-(4-(Methylsulfonyl)phenyl)thiophen-2-yl)isobenzofuran-1(3*H*)-one (60).** Compound **5** was reacted with 4-(methanesulfonyl)phenylboronic acid according to general procedure B. The crude product was purified by flash column chromatography on silica gel (CH_2_Cl_2_ as eluant), followed by trituration with CH_2_Cl_2_/Et_2_O to give the title compound **60** as a pale yellow solid (50%), mp (CH_2_Cl_2_/Et_2_O) 278-280 ^o^C. ^1^H NMR [(CD_3_)_2_SO] δ 8.03 (s, 1 H), 7.99 (s, 4 H), 7.95 (d, *J* = 7.9 Hz, 1 H), 7.90 (d, *J* = 7.9 Hz, 1 H), 7.84 (s, 2 H), 5.46 (s, 2 H), 3.25 (s, 3 H). LRMS (APCI^+^) calcd for C_19_H_15_O_4_S_2_ 371 (MH^+^), found 371. Anal. (C_19_H_14_O_4_S_2_) C, H.

**3-(5-(1-Oxo-1,3-dihydroisobenzofuran-5-yl)thiophen-2-yl)benzenesulfonamide (61).** Compound **5** was reacted with 3-aminosulfonylbenzene boronic acid according to general procedure B. The crude product was purified by flash column chromatography on silica gel (5% MeOH/CH_2_Cl_2_ as eluant), followed by trituration with Et_2_O/CH_2_Cl_2_ to give the title compound **61** as a yellow solid (48%), mp (CH_2_Cl_2_/Et_2_O) 273-276 ^o^C. ^1^H NMR [(CD_3_)_2_SO] δ 8.13 (t, *J* = 1.6 Hz, 1 H), 8.03 (s, 1 H), 7.92-8.00 (m, 2 H), 7.90 (d, *J* = 8.1 Hz, 1 H), 7.83 (d, *J* = 3.9 Hz, 1 H), 7.80 (d, *J* = 8.0 Hz, 1 H), 7.72 (d, *J* = 3.9 Hz, 1 H), 7.66 (t, *J* = 7.8 Hz, 1 H), 7.45 (br s, 2 H), 5.46 (s, 2 H). LRMS (APCI^+^) calcd for C_18_H_14_NO_4_S_2_ 372 (MH^+^), found 372. Anal. (C_18_H_13_NO_4_S_2_.0.5H_2_O) C, H, N.

***N*-*tert*-Butyl-4-(5-(1-oxo-1,3-dihydroisobenzofuran-5-yl)thiophen-2-yl)benzenesulfonamide (62).** Compound **5** was reacted with 4-(*t*-butylamino)sulfonylphenylboronic acid pinacol ester according to general procedure B. The crude product was purified by flash column chromatography on silica gel (CH_2_Cl_2_ as eluant), followed by trituration with CH_2_Cl_2_/Et_2_O to give the title compound **62** as a pale yellow solid (48%), mp (CH_2_Cl_2_/Et_2_O) 255-257 ^o^C. ^1^H NMR [(CD_3_)_2_SO] δ 8.02 (s, 1 H), 7.86-7.96 (m, 6 H), 7.83 (d, *J* = 3.9 Hz, 1 H), 7.78 (d, *J* = 3.9 Hz, 1 H), 7.56 (s, 1 H), 5.46 (s, 2 H), 1.12 (s, 9 H). LRMS (APCI^-^) calcd for C_22_H_20_NO_4_S_2_ 426 (M-H), found 426. Anal. (C_22_H_21_NO_4_S_2_) C, H, N.

***N*-(4-(5-(1-Oxo-1,3-dihydroisobenzofuran-5-yl)thiophen-2-yl)phenyl)methanesulfonamide (63).** Compound **5** was reacted with 4-methanesulfonylaminophenylboronic acid according to general procedure B. The desired product precipitated out of the reaction mixture, was collected by filtration and washed well with water and 10% MeOH/CH_2_Cl_2_ to give the title compound **63** as a pale green solid (55%), mp (CH_2_Cl_2_/Et_2_O) >300 ^o^C. ^1^H NMR [(CD_3_)_2_SO] δ 9.90 (s, 1 H), 7.97 (s, 1 H), 7.91 (dd, *J* = 1.4, 8.0 Hz, 1 H), 7.87 (d, *J* = 7.9 Hz, 1 H), 7.75 (d, *J* = 3.9 Hz, 1 H), 7.68 (d, *J* = 8.6 Hz, 2 H), 7.53 (d, *J* = 3.9 Hz, 1 H), 7.26 (d, *J* = 8.6 Hz, 2 H), 5.44 (s, 2 H), 3.02 (s, 3 H). HRMS (FAB^+^) calcd for C_19_H_16_NO_4_S_2_ 386.0521 (MH^+^), found 386.0513.

**5-(5-(1-Oxo-1,3-dihydroisobenzofuran-5-yl)thiophen-2-yl)picolinamide (67).** Compound **5** (1.00 g, 3.39 mmol), bis(pinacolato)diboron (2.58 g, 10.2 mmol) and KOAc (1.66 g, 16.7 mmol) were weighed into a flask and suspended in DMSO (25 mL). The mixture was heated to 90 ^o^C for 4 h and then allowed to cool to room temperature. All solvent was removed under reduced pressure and the resulting solid dissolved in CH_2_Cl_2_ (100 mL) and washed with water (50 mL) and brine (50 mL). The CH_2_Cl_2_ layer was dried (Na_2_SO_4_), filtered, and the solvent removed under reduced pressure. The crude product was purified by flash column chromatography on silica gel (20% EtOAc/hexanes as eluant), followed by trituration with Et_2_O to afford crude compound **66** as a cream solid (approx. 70% pure by ^1^H NMR). This material was used directly in the next step without further purification.

Compound **65** was prepared from **64** using a procedure from a literature reference^13^ and reacted with **66** according to general procedure B. After 1.5 h the mixture was cooled to room temperature and partitioned between water and CH_2_Cl_2_. Both phases were filtered and the combined crude solid was suspended in 10% MeOH/CH_2_Cl_2_ and stirred for 10 min. The solid was filtered and purified by flash column chromatography on silica gel (10-20% acetone/CH_2_Cl_2_ as eluant) to give the title compound **67** as a pale yellow solid (14%), mp 304-307 ^o^C. ^1^H NMR [(CD_3_)_2_SO] δ 9.00 (dd, *J* = 2.3, 0.7 Hz, 1 H), 8.29 (dd, *J* = 8.2, 2.3 Hz, 1 H), 8.06-8.12 (dd, *J* = 8.2, 0.7 Hz, 2 H), 8.04 (s, 1 H), 7.96 (dd, *J* = 8.1, 1.5 Hz, 1 H), 7.91 (d, *J* = 8.4 Hz, 1 H), 7.89 (d, *J* = 3.9 Hz, 1 H), 7.87 (d, *J* = 3.9 Hz, 1 H), 7.66 (br s, 1 H), 5.47 (s, 2 H). LRMS (APCI^+^) calcd for C_18_H_12_N_2_O_3_S 337 (MH^+^), found 337. Anal. (C_18_H_12_N_2_O_3_S.0.25H_2_O) C, H, N.

**5-(5-(Pyridin-4-yl)thiophen-2-yl)isobenzofuran-1(3*H*)-one (70).** Compound **69** was prepared according to a literature procedure^18^ and was reacted with **4** according to general procedure A. Purification was carried out by preparative-HPLC [Agilent Zorbax SB C18 column eluting with 10-90% MeCN/45 mM NH_4_HCO_2_] to give the title compound **70** as a yellow solid (27%), mp 260-261 ^o^C. ^1^H NMR [(CD_3_)_2_SO] δ 8.62 (d, *J* = 6.2 Hz, 2 H), 8.04 (s, 1 H), 7.96 (dd, *J* = 8.1, 1.5 Hz, 1 H), 7.93 (d, *J* = 3.7 Hz, 1 H), 7.91 (d, *J* = 8.3 Hz, 1 H), 7.86 (d, *J* = 3.9 Hz, 1 H), 7.71 (d, *J* = 6.2 Hz, 2 H), 5.47 (s, 2 H). HRMS (ESI^+^) calcd for C_17_H_12_NO_2_S 294.0583 (MH^+^), found 294.0589.

**5,5'-(Thiophene-2,5-diyl)diisobenzofuran-1(3*H*)-one (71).** Compound **5** was reacted with **28** according to general procedure B. The resulting crude solid was triturated with 10% MeOH/CH_2_Cl_2_ to give the title compound **71** as a yellow solid (77%), mp (MeOH/CH_2_Cl_2_) >300 ^o^C. ^1^H NMR [(CD_3_)_2_SO] δ 8.03 (s, 2 H), 7.96 (dd, *J* = 7.9, 1.3 Hz, 2 H), 7.91 (d, *J* = 7.9 Hz, 2 H), 7.85 (s, 2 H), 5.47 (s, 4 H). LRMS (APCI^-^) calcd for C_20_H_11_O_4_S 347 (M-H), found 347. Anal. (C_20_H_12_O_4_S.0.5H_2_O) C, H.

**1.1.1. Table 1: Elemental analysis results for target compounds**

|  |  | Calcd | | | Found | | |
| --- | --- | --- | --- | --- | --- | --- | --- |
| **Compound** |  | C | H | N | C | H | N |
| **6** | C_18_H_12_O_2_S | 73.9 | 4.1 |  | 74.0 | 4.1 |  |
| **7** | C_19_H_14_O_2_S.0.25H_2_O | 73.4 | 4.7 |  | 73.5 | 4.7 |  |
| **8** | C_19_H_14_O_2_S.0.25H_2_O | 73.4 | 4.7 |  | 73.4 | 4.8 |  |
| **9** | C_19_H_14_O_2_S.0.25H_2_O | 73.4 | 4.7 |  | 73.6 | 4.7 |  |
| **10** | C_18_H_11_ClO_2_S | 66.2 | 3.4 |  | 66.1 | 3.6 |  |
| **11** | C_18_H_11_ClO_2_S.0.25H_2_O | 65.3 | 3.5 |  | 65.2 | 3.7 |  |
| **12** | C_18_H_11_ClO_2_S | 66.2 | 3.4 |  | 66.1 | 3.5 |  |
| **13** | C_19_H_14_O_3_S.0.5H_2_O | 68.9 | 4.6 |  | 68.9 | 4.3 |  |
| **14** | C_19_H_14_O_3_S | 70.8 | 4.4 |  | 70.9 | 4.4 |  |
| **15** | C_19_H_14_O_3_S.0.25H_2_O | 69.8 | 4.5 |  | 70.1 | 4.5 |  |
| **18** | C_18_H_12_O_3_S.0.5H_2_O | 68.1 | 4.1 |  | 67.7 | 3.9 |  |
| **19** | C_18_H_12_O_3_S.0.25H_2_O | 69.1 | 4.0 |  | 69.0 | 3.9 |  |
| **20** | C_19_H_11_NO_2_S.0.25H_2_O | 70.9 | 3.6 | 4.4 | 70.9 | 3.4 | 4.3 |
| **21** | C_19_H_11_NO_2_S | 71.9 | 3.5 | 4.4 | 71.4 | 3.6 | 4.4 |
| **22** | C_19_H_13_NO_3_S.0.5H_2_O | 66.3 | 4.1 | 4.1 | 66.4 | 3.9 | 3.9 |
| **23** | C_19_H_13_NO_3_S.0.75H_2_O | 65.4 | 4.2 | 4.0 | 65.6 | 4.0 | 4.2 |
| **23a** | C_19_H_14_N_2_O_2_S.0.75H_2_O | 65.6 | 4.5 | 8.1 | 65.7 | 4.2 | 7.9 |
| **23b** | C_20_H_16_N_2_O_2_S.0.25H_2_O | 68.1 | 4.7 | 7.9 | 67.7 | 4.7 | 7.8 |
| **29** | C_20_H_15_NO_3_S.0.5H_2_O | 67.0 | 4.5 | 3.9 | 70.0 | 4.3 | 3.9 |
| **30** | C_21_H_17_NO_3_S.0.25H_2_O | 68.6 | 4.8 | 3.9 | 68.8 | 4.7 | 3.9 |
| **31** | C_24_H_22_N_2_O_3_S | 68.9 | 5.3 | 6.7 | 68.5 | 5.1 | 6.5 |
| **32** | C_24_H_24_N_2_O_3_S.0.75H_2_O | 66.4 | 5.9 | 6.5 | 66.3 | 5.6 | 6.2 |
| **39** | C_21_H_16_O_4_S.0.25H_2_O | 68.4 | 4.5 |  | 68.5 | 4.4 |  |
| **40** | C_21_H_16_O_5_S | 66.3 | 4.2 |  | 66.7 | 4.1 |  |
| **48** | C_19_H_13_NO_4_S.0.5H_2_O | 63.3 | 3.9 | 3.9 | 63.1 | 3.9 | 3.8 |
| **49** | C_25_H_24_N_2_O_5_S.0.5H_2_O | 63.4 | 5.3 | 5.9 | 63.6 | 5.1 | 5.8 |
| **51** | C_21_H_17_NO_5_S.0.25H_2_O | 63.1 | 4.1 | 3.5 | 63.2 | 4.4 | 3.3 |
| **55** | C_19_H_14_N_2_O_3_S | 65.1 | 4.0 | 8.0 | 65.1 | 4.2 | 7.7 |
| **56** | C_25_H_25_N_3_O_4_S | 64.8 | 5.4 | 9.1 | 64.6 | 5.4 | 9.1 |
| **58** | C_19_H_14_O_2_S_2_.0.25H_2_O | 66.5 | 4.3 |  | 66.8 | 4.2 |  |
| **59** | C_19_H_14_O_3_S | 70.8 | 4.4 |  | 71.0 | 4.4 |  |
| **60** | C_19_H_14_O_4_S_2_ | 61.6 | 3.8 |  | 61.5 | 3.8 |  |
| **61** | C_18_H_13_NO_4_S_2_.0.5H_2_O | 56.8 | 3.7 | 3.7 | 56.7 | 3.5 | 3.5 |
| **62** | C_22_H_21_NO_4_S_2_ | 61.8 | 4.9 | 3.3 | 61.5 | 5.0 | 3.2 |
| **67** | C_18_H_12_N_2_O_3_S.0.25H_2_O | 63.4 | 3.7 | 8.2 | 63.2 | 3.7 | 7.7 |
| **71** | C_20_H_12_O_4_S.0.5H_2_O | 67.2 | 3.7 |  | 67.3 | 3.6 |  |

**1.1.2. Table 2: Elemental analyses for selected intermediates**

|  |  | Calcd | | | Found | | |
| --- | --- | --- | --- | --- | --- | --- | --- |
| **Compound** |  | C | H | N | C | H | N |
| **5** | C_12_H_7_BrO_2_S.0.1EtOAc | 49.3 | 2.6 |  | 48.9 | 2.4 |  |
| **26** | C_13_H_12_BrNOS | 50.3 | 3.9 | 4.5 | 50.3 | 3.8 | 4.4 |
| **38** | C_13_H_11_BrO_3_S | 47.7 | 3.4 |  | 47.6 | 3.5 |  |

**1.1.3 Table 3: HRMS and HPLC results for target compounds**

|  | HRMS |  |  | HPLC |
| --- | --- | --- | --- | --- |
| **Compound** | Formula | Calcd. | Found |  |
| **16** | C_15_H_12_N_2_O_2_S_2_ (M^+^) | 307.0667 | 307.0669 | 98.1% |
| **17** | C_18_H_13_NO_2_S (M^+^) | 307.0667 | 307.0673 | 98.4% |
| **46** | C_19_H_14_NO_4_S (MH^+^) | 352.0644 | 352.0647 | 95.6% |
| **50** | C_23_H_23_N_2_O_4_S (MH^+^) | 423.1379 | 423.1381 | 91.1% |
| **52** | C_22_H_20_NO_5_S (MH^+^) | 410.1062 | 410.1071 | 99.9% |
| **53** | C_21_H_17_N_2_O_5_S (MH^+^) | 409.0858 | 409.0850 | 99.0% |
| **54** | C_22_H_19_N_2_O_5_S (MH^+^) | 423.1015 | 423.1010 | 95.0% |
| **57** | C_23_H_24_N_3_O_3_S (MH^+^) | 422.1538 | 422.1541 | 90.0% |
| **63** | C_19_H_16_NO_4_S_2_ (MH^+^) | 386.0521 | 386.0513 | 98.0% |
| **70** | C_17_H_12_NO_2_S (MH^+^) | 294.0583 | 294.0589 | 99.8% |

**1.1.4 Table 4: Solubility data for selected compounds**

| **Compound** | Inhibition of Jurkat Cell Lysis IC_50_ (μM) | Solubility  (water, μg/mL) |
| --- | --- | --- |
| **48** | 0.67 | 0.023 |
| **51** | 3.31 | 15.7 |
| **53** | 2.65 | 4.7 |
| **55** | 1.20 | 9.4 |
| **67** | 0.92 | 0.204 |

**1.2. Biology**

**1.2.1. Inhibition of perforin-mediated lysis of sheep red blood cells**

As reported previously,^8^ compound **1** was identified from screening a commercial library of 100,000 compounds for the ability to reproducibly inhibit perforin-mediated lysis of SRBC at a compound concentration of 100 μM.

**1.2.2. Inhibition of perforin-mediated lysis of Jurkat cells**

The ability of the compounds to inhibit the lysis of nucleated (Jurkat T lymphoma) cells in the presence of 0.1% BSA, as measured by release of ^51^Cr was measured. Jurkat target cells were labelled by incubation in medium with 100 μCi ^51^Cr for one hour. The cells were then washed three times to remove unincorporated isotope and re-suspended at 1 x 10^5^ cells per mL in RPMI buffer supplemented with 0.1% BSA. Each test compound was pre-incubated to concentrations of 20, 10, 5, 2.5 and 1.25 μM with recombinant perforin for 30 min with DMSO as a negative control. ^51^Cr labelled Jurkat cells were then added and cells were incubated at 37 °C for 4 h. The supernatant was collected and assessed for its radioactive content on a gamma counter (Wallac Wizard 1470 automatic gamma counter). Each data point was performed in triplicate and an IC_50_ was calculated from the range of concentrations described to above. Compounds with an IC_50_ <1 μM were titrated down to lower concentrations in the same manner as above, to determine an accurate IC_50_. Compounds with IC_50_ < 1 μM were titrated to lower concentrations in the same manner as above, to determine an accurate IC_50_.

**1.2.3. KHYG-1 cytotoxicity assay**

KHYG-1 cells were washed and re-suspended in RPMI + 0.1% BSA at 4 × 10^5^ cells/mL, and an amount of 50 μL of KHYG-1 cells was dispensed to each well of a 96-well V-bottom plate. RPMI (50 μL) + 0.1% BSA or 10% (final concentration) of serum was added to each well. Then test compounds were added to KHYG-1 cells at various concentrations up to 20 μM and incubated at room temperature for 20 min. 1 × 10^6^ K562 target cells were labelled with 75 μCi ^51^Cr in 200 μL of RPMI for 90 min at 37 °C. Cells were washed as described above and re-suspended in 5 mL of RPMI + 0.1% BSA. An amount of 50 μL of ^51^Cr labelled K562 leukaemia target cells was added to each well of the KHYG-1 plate (effector/target 2:1) and incubated at 37 °C for 4 h. ^51^Cr release was assayed using a Skatron harvesting press and radioactivity estimated on a Wallac Wizard 1470 automatic γ counter (Turku, Finland). The percentage of specific cytotoxicity was calculated using the following formula:

% specific lysis = (experimental release - spontaneous release) x 100

(maximum release - spontaneous release)

and expressed as the mean of triplicate assays ± standard error of the mean.

**1.2.4. Toxicity to KHYG-1 NK cells**

The toxicity assay was carried out in exactly the same manner as the killing assay above, but instead of adding the labelled K562 target cells, 100 μL of RPMI 0.1% BSA was added. Cells were incubated for 4 h at 37 °C and then washed ×3 in RPMI + 0.1% BSA. Cells were then re-suspended in 200 μL of complete medium and incubated for 18−24 h at 37 °C. Trypan blue was added to each well. Viable (clear) cells and total (clear + blue) cells were counted, and the percentage of viable cells was calculated compared to DMSO treated cell control (% viability).

**2. Plasma stability studies**

*Incubation method -* Mouse (CD-1, male) and human (pooled) plasma pH was adjusted to 7.4 with either 1M phosphoric acid or sodium hydroxide. Plasma was spiked with 100 uM of test compounds and divided into two parts; one part was immediately transferred to -80 °C (control), and the second part was incubated at 37 °C at 24 h.

At the end of the study duration, samples were transferred to -80 °C until ready to analyse by HPLC-DAD. On the day of analysis, control and incubated samples were thawed on wet ice and mixed.

*Sample treatment for HPLC* **-** 20 uL of samples were mixed with 80 uL (1:4 v/v) ice cold acetonitrile to precipitate the plasma proteins, centrifuged at 13,000 rpm for 5 min to obtain the clear supernatant. The supernatant was diluted with 0.01% formic acid– water (1:2 v/v) and 50 uL was injected onto the HPLC system (Agilent).

*HPLC column, conditions & calculation -*

Column: Column Alltima C8, 5µ, 150 mm x 2.1 mm, and a pre-column guard Alltima C8, 5 µ 7.5 mm x 2.1 mm (both from Grace Discovery Sciences, USA ).

Conditions: Solvent flow rate = 0.4 mL/min; total run time = 20 min run time + 2 min post time; aqueous phase = 45 mM FB, pH 4.5; organic phase = 80% acetonitrile + 20% water.

Gradient time table:

| Time (min) | % Organic |
| --- | --- |
| 0 | 20 |
| 0.2 | 20 |
| 8.0 | 100 |
| 16.0 | 20 |
| 20 | 20 |

Calculation: The % loss was calculated based on peak area using the following formula: (peak area time “0”- peak area time “24h”)/peak area time “0” multiplied by 100. % Remaining = 100 - % loss.

**3. References (numbered as according to the manuscript)**

8. Spicer, J. A.; Lena, G.; Lyons, D. M.; Huttunen, K. M.; Miller, C. K.; O'Connor, P. D.; Bull, M.; Helsby, N. A.; Jamieson, S. M. F.; Denny, W. A.; Ciccone, A.; Browne, K. A.; Lopez, J. A.; Rudd-Schmidt, J.; Voskoboinik, I.; Trapani, J. *J. Med. Chem.* **2013**, *56*, 9542.

13. Klapars, A.; Buchwald, S. L. *J. Am. Chem. Soc.* **2002**, *124*(50), 14844.

14. Kumar, N.; Nayyer, S.; Mitu, G. Patent WO 2006103550 A1, 5 October **2006**.

15. Kobayashi, K.; Sugie, A.; Takahashi, M.; Masui, K.; Mori, A. *Org. Lett.* **2005**, *7*(22), 5083.

16. Spicer, J. A.; Denny, W. A.; Miller, C. K.; O'Connor, P. D.; Huttunen, K.; Trapani, J. A.; Hill, G.; Alexander, K. Patent WO 2014028968 A1, 27 February, **2014**.

17. Ernst, G.; Frietze, W.; Jacobs, R.; Phillips, E. Patent WO 2005061510 A1, 7 July **2005**.

19. Effenberger, F.; Endtner, J. M.; Miehlich, B.; Münter, J. S. R.; Vollmer, M. S. *Synthesis.* **2000**, *9*, 1229.
